# Associations of mental health symptoms and triglyceride-glucose index with incident cardiovascular disease: a cohort study from the UK Biobank

**DOI:** 10.3389/fendo.2026.1874050

**Published:** 2026-06-24

**Authors:** Tianci Yao, Ying Zhu, Hongyu Yan, Qinmei Ke

**Affiliations:** 1 Department of Endocrinology, Yueyang Central Hospital, Yueyang, China; 2 Department of Geriatrics, Chongqing Hospital, Union Hospital, Tongji Medical College, Huazhong University of Science and Technology, Chongqing, China; 3 Department of Geriatrics, Union Hospital, Tongji Medical College, Huazhong University of Science and Technology, Wuhan, China

**Keywords:** 4-item patient health questionnaire, cardiovascular disease, insulin resistance, mental health symptoms, triglyceride-glucose index

## Abstract

**Background:**

Mental health symptoms often coexist with insulin resistance (IR), and they are independently associated with incident cardiovascular disease (CVD). However, it remains unclear whether mitigating IR can reduce the risk of incident CVD in populations with mental health symptoms.

**Methods:**

This study included 250,716 adults from the UK Biobank free of prevalent CVD at baseline. IR was reflected by the triglyceride-glucose (TyG) index. Mental health symptoms were assessed by the 4-item Patient Health Questionnaire (PHQ-4) scores, categorized as no symptoms (0), mild symptoms (1), and clear symptoms (≥ 2). We used Cox proportional hazards models to assess the independent and joint associations of TyG and PHQ-4 with incident CVD and myocardial infarction (MI). The multiplicative and additive interactions were assessed between TyG tertiles and PHQ-4 status.

**Results:**

During a median follow-up of 13.6 years, 22,867 incident CVD and 7,649 incident MI cases were recorded. Compared to participants with PHQ-4 = 0, those with PHQ-4 ≥ 2 had higher risks of incident CVD (hazard ratio [HR], 1.35; 95% confidence interval [CI], 1.31–1.39) and MI (1.30, 1.23–1.37), and the proportions mediated by TyG were 5.3% (4.5%–6.3%) and 9.3% (7.4%–12.5%), respectively, whereas no significant mediation effect was observed in participants with PHQ-4 = 1. Significant multiplicative (HR for interaction, 1.11; 95%CI, 1.02–1.19) and additive interactions (relative excess risk due to interaction [RERI], 0.25; 95%CI, 0.15–0.35) were found between TyG tertile 3 and PHQ-4 ≥ 2 on incident CVD, and additive interaction (RERI, 0.33; 95%CI, 0.14–0.52) was seen on incident MI. Those with PHQ-4 ≥ 2 and TyG tertile 3 had the highest risks of incident CVD (1.80, 1.71–1.90) and incident MI (2.17, 1.97–2.38).

**Conclusions:**

Clear mental health symptoms (PHQ-4 score ≥ 2) were associated with higher risk of incident CVD, with IR reflected by TyG index partially and modestly mediating the association, suggesting that targeting IR may help attenuate cardiovascular risk among populations with clear mental health symptoms. Our findings support integrated interventions to reduce the burden of CVD, particularly in individuals with clear mental health symptoms who are more vulnerable to IR.

## Introduction

Cardiovascular disease (CVD) remains one of the leading causes of death and disability worldwide, imposing substantial health and economic burdens (1, 2). Depression and anxiety are the most prevalent and disabling mental health symptoms (3), which often coexist with incident CVD (4–6). The risk of incident CVD is also significantly higher among people with these mental health conditions compared to the general population (6). Given the rising prevalence of these mental health symptoms (7), clarifying the biological pathways linking them to CVD is critical for identifying effective preventive strategies.

Mental health symptoms frequently coexist with metabolic disturbances, particularly insulin resistance (IR) (8), which is a key mechanism of multiple metabolic diseases and also recognized as a significant risk factor for CVD (9, 10). Evidence suggests shared pathophysiological mechanisms between IR and mental health symptoms, including dysregulation of the hypothalamic-pituitary-adrenal axis, chronic low-grade inflammation and alterations in the gut microbiota and neurotransmitter systems, indicating a potential interplay where each condition may influence the onset of the other (8, 11, 12). Nevertheless, the practical evaluation of IR in large populations remains challenging. The gold standard for IR, the hyperinsulinemic-euglycemic clamp test, is not feasible for large-scale studies due to its time-consuming and expensive procedure (13). Homeostasis model assessment of IR is a commonly used surrogate but it has limited value in subjects receiving insulin treatment or without functioning beta cells (14). To address these limitations, the triglyceride-glucose (TyG) index, derived from fasting triglycerides and glucose, has emerged as a simple and reliable surrogate of IR that has potential clinical utility in predicting cardiovascular risk (15, 16).

Studies have shown that a single metabolic factor may mediate the connection between depression or anxiety and incident CVD (17–19). However, because depression and anxiety often coexist (20), a measure of comorbid mental health burden may better capture the real-world impact on cardiovascular outcomes. The 4-item Patient Health Questionnaire (PHQ-4) is an ultra-brief self-report questionnaire consisting of a 2-item depression scale and a 2-item anxiety scale, which reliably provides a comprehensive assessment of mental health in the general population (21). To our knowledge, few studies have evaluated whether IR mediates the relationship between mental health symptoms and CVD, or whether mental health symptoms and IR interact synergistically to accelerate CVD progression. By integrating IR with mental health problems, this work aims to elucidate these pathophysiological pathways and inform dual-targeted interventions for CVD prevention.

This study addresses these gaps by examining, in a large-scale prospective cohort, the independent and joint associations of mental health symptoms and IR with incident CVD and myocardial infarction (MI). We further quantify the mediating role of IR in the relationship between mental health symptoms and CVD risk, and assess potential interactions between mental health symptoms and IR.

## Materials and methods

### Study design and population

The UK Biobank is a large prospective cohort study that recruited over 500,000 participants aged 37 to 73 years between March 2006 and October 2010. Details of data collection were described previously (22). The UK Biobank obtained ethical approval from the National Health Service and the National Research Ethics Service (reference 11/NW/0382), and all participants provided written informed consent.

Among the 502,356 participants, we excluded those with missing data on the TyG index (n = 73,236) and PHQ-4 (n = 45,212) at baseline, those with missing information on covariates (n = 116,826), and those diagnosed with CVD at baseline (n = 16,366), leaving 250,716 participants in the final analysis (**Supplementary Figure 1**). The diagnosis of prevalent CVD was obtained through self-reported disease history and linked hospital admissions data (ICD-9: 410–414, 428, 430–434, 436; ICD-10: I20-I25, I50, I60-I64).

### Assessment of TyG index and mental health symptoms

The TyG index was calculated as ln [fasting plasma glucose (mg/dL) × triglyceride (mg/dL)/2] (23) Triglycerides and fasting plasma glucose were quantified using clinical chemistry (Beckman Coulter AU5800; LOCATION of manufacturer) on peripheral venous blood samples collected at baseline following validated UK Biobank protocols (24).

To screen for mental health symptoms, we used the PHQ-4, a brief self-report instrument that is widely recognized for its reliability and validity in the general population (21). The PHQ-4 assesses the presence of mental health symptoms over the past two weeks using a 4-point scale: 0 (not at all), 1 (several days), 2 (more than half the days), and 3 (nearly every day) (25). This assessment encompassed the 2-item Patient Health Questionnaire for depression and the other 2-item Generalized Anxiety Disorder Screener for anxiety (25). A large-scale European study has recommended that a total PHQ-4 score of ≥ 2 serves as optimal for detecting depressive or anxiety disorder (26). We interpreted PHQ-4 scores as indicating no symptoms (0), mild symptoms (1), and clear symptoms (≥ 2). Mild symptoms (PHQ-4 score =1) were defined as the presence of any one symptom—depressed mood, loss of interest, nervousness, or fatigue—occurring on several days, whereas clear symptoms (PHQ-4 score ≥2) were defined as any one symptom on more than half the days, any two symptoms on several days, or more severe disturbances within the past two weeks.

### Assessment of covariates

The covariates were obtained through questionnaires, including age, sex, race, educational level, employment status, socioeconomic status (Townsend Deprivation Index), smoking status, alcohol consumption, physical activity, diet, and self-reported medication use (antihypertensive drugs, lipid-lowering drugs, and insulin). Educational level was divided into two categories: college or university degree, and others (including A levels/AS levels or equivalent, O levels/GCSEs or equivalent, CSEs or equivalent, NVQ or HND or HNC or equivalent, other professional qualifications) (27). Employment status was classified into two categories: employed and retired or unemployed (28). The Townsend deprivation index is a composite measure of socioeconomic deprivation. Smoking status was categorized as current smoking, previous smoking, and never smoking. Alcohol intake was quantified as average grams per day based on consumption frequency and weekly or monthly volumes of six beverage types (29). Physical activity was categorized into <150 minutes/week or ≥ 150 minutes/week of moderate-to-vigorous physical activity using the guideline-recommended threshold (30). Dietary quality was assessed based on dietary recommendations for cardiovascular health, with adherence to at least half of the 10 recommended food components defined as a healthy diet (31).

### Ascertainment of outcomes

The outcomes of this study included incident CVD (ICD-10 codes: I20–I25, I50, I60–I64) and MI (ICD-10 codes: I21–I23, I24.1, I25.2), which were regularly ascertained through linkage with hospital admission records and death registry data. The hospital registry-based follow-up ended on October 31, 2022, in England; August 31, 2022, in Scotland; and May 31, 2022, in Wales.

### Statistical analysis

For longitudinal analyses, person-years were calculated from the date of recruitment to the date of diagnosis or death, loss to follow-up, or the end of follow-up, whichever occurred first. We assessed the associations of the PHQ-4 score and TyG index with risks of incident CVD and MI using Cox proportional hazards models adjusted for age, sex, race, educational level, employment status, Townsend Deprivation Index, smoking status, alcohol consumption, physical activity, diet, and self-reported medication use. The proportional hazards assumption was tested by using a likelihood ratio test comparing models with and without time-dependent exposure, and we found no significant deviation from the assumption. Moreover, the dose-response associations of the PHQ-4 score or TyG index with risks of incident CVD and MI were examined using restricted cubic spline (RCS) regression.

In the mediation analysis, we examined whether the associations of elevated PHQ-4 scores with increased risks of incident CVD and MI were mediated by the TyG index. We constructed two regression models, a logistic model, to regress the outcome (incident CVD and MI) on the exposure (PHQ-4 score) and the mediator (TyG index), and a linear model, to regress the mediator on the exposure, with adjustment for potential confounders. We integrated these two regressions to estimate the proportion of mediating effect, and 95% CIs were obtained via bootstrapping.

We performed stratified analyses to explore the associations of the TyG index with incident CVD and MI among various subgroups of PHQ-4 status. Using multiplicative and additive interaction analyses, we evaluated whether the associations between TyG index and health outcomes differed by PHQ-4 status. Multiplicative interaction was tested by including a cross-product term between the TyG index and PHQ-4 status in adjusted multivariable models, with the hazard ratio and its 95% confidence interval used to evaluate the interaction. Additive interaction was assessed using the relative excess risk due to interaction (RERI) and corresponding 95% confidence intervals. Finally, we assessed the joint association of TyG index and PHQ-4 status with health outcomes by dividing participants into nine groups based on the combined categories of TyG index and PHQ-4 status.

To test the robustness and potential variations in different subgroups, we repeated all analyses stratified by age (< 65 and ≥ 65), sex (male and female), and race (white people and non-white people).

We conducted several sensitivity analyses. First, we excluded events that occurred within the first two years after recruitment to reduce potential reverse causation. Second, considering the possible non-linear associations of age with health outcomes, we additionally included quadratic terms of age in the model. Third, we used multiple imputations with chained equations to impute all missing independent variables and covariates to test the influence of missing values. Specifically, the imputation model included the TyG index, PHQ-4, and all major covariates used in the fully adjusted models. Twenty imputed datasets (*m* = 20) were generated using chained equations with a fixed random seed (seed = 1234) to ensure reproducibility. Fourth, to explore the specific PHQ-4 threshold that aligns with our study objectives, we tested alternative thresholds for PHQ-4 status, defining no symptoms as a score of 0, mild symptoms as 1–2, and clear symptoms as ≥ 3.

All analyses were performed using R statistical software version 4.3.2. A two-sided test with *P* < 0.05 was considered statistically significant.

## Results

### Baseline characteristics

Baseline characteristics by PHQ-4 status are shown in **Table 1**. Among 250,716 participants (median age 57.0 years; 46.6% men), 41.3% had PHQ-4 = 0, 24.8% had PHQ-4 = 1, and 33.9% had PHQ-4 ≥ 2. Those with PHQ-4 ≥ 2 were more often younger, female, socioeconomically deprived, less educated, and employed, with higher prevalence of smoking, physical inactivity, unhealthy diet, and insulin use. Participants with higher TyG levels were more likely to have higher PHQ-4 scores (**Supplementary Table 1**). Excluded participants tended to be older, female, less educated, more deprived, unemployed, and had higher PHQ-4 and TyG levels (**Supplementary Table 2**).

**Table 1:** Baseline characteristics of the study population according to PHQ-4 status.

Variables	PHQ-4 = 0 (N = 103497)	PHQ-4 = 1 (N = 62183)	PHQ-4 ≥ 2 (N = 85036)	*P* value
Age, years	59.0 [52.0, 64.0]	57.0 [49.0, 63.0]	55.0 [48.0, 61.0]	<0.001
Sex, n (%)				<0.001
Female	49637 (48.0%)	35103 (56.5%)	49050 (57.7%)	
Male	53860 (52.0%)	27080 (43.5%)	35986 (42.3%)	
Race, n (%)				<0.001
Asian	1273 (1.2%)	602 (1.0%)	1725 (2.0%)	
Black	1019 (1.0%)	464 (0.7%)	1245 (1.5%)	
Other	907 (0.9%)	583 (0.9%)	1281 (1.5%)	
White	100298 (96.9%)	60534 (97.3%)	80785 (95.0%)	
Townsend deprivation index, n (%)^a^				<0.001
Least deprived	24838 (24.0%)	14165 (22.8%)	16656 (19.6%)	
Intermediate deprived	64532 (62.4%)	38722 (62.3%)	51027 (60.0%)	
Most deprived	14127 (13.6%)	9296 (14.9%)	17353 (20.4%)	
Educational level, n (%)^b^				<0.001
Others	49797 (48.1%)	28636 (46.1%)	43013 (50.6%)	
College or university degree	53700 (51.9%)	33547 (53.9%)	42023 (49.4%)	
Employment status, n (%)				<0.001
Employed	57439 (55.5%)	39444 (63.4%)	54715 (64.3%)	
Retired/unemployed	46058 (44.5%)	22739 (36.6%)	30321 (35.7%)	
Self-reported use of antihypertensive drugs, n (%)				0.671
No	85826 (82.9%)	51640 (83.0%)	70641 (83.1%)	
Yes	17671 (17.1%)	10543 (17.0%)	14395 (16.9%)	
Self-reported use of lipid-lowering drugs, n (%)				0.005
No	89688 (86.7%)	54130 (87.0%)	74093 (87.1%)	
Yes	13809 (13.3%)	8053 (13.0%)	10943 (12.9%)	
Self-reported use of insulin, n (%)				<0.001
No	102815 (99.3%)	61646 (99.1%)	84096 (98.9%)	
Yes	682 (0.7%)	537 (0.9%)	940 (1.1%)	
Smoking status, n (%)				<0.001
Never	61143 (59.1%)	36000 (57.9%)	47034 (55.3%)	
Previous	35748 (34.5%)	21443 (34.5%)	28564 (33.6%)	
Current	6606 (6.4%)	4740 (7.6%)	9438 (11.1%)	
Alcohol intake, g/day^c^	11.4 [3.3, 21.4]	11.4 [2.0, 20.0]	11.4 [2.0, 20.0]	<0.001
Physical activity, n (%)^d^				<0.001
<150 min/wk MVPA	32166 (31.1%)	23320 (37.5%)	34607 (40.7%)	
≥150 min/wk MVPA	71331 (68.9%)	38863 (62.5%)	50429 (59.3%)	
Diet^e^				<0.001
<5 health diet	84104 (81.3%)	52274 (84.1%)	71853 (84.5%)	
≥5 health diet	19393 (18.7%)	9909 (15.9%)	13183 (15.5%)	
TyG	8.7 [8.3, 9.0]	8.7 [8.3, 9.0]	8.7 [8.3, 9.1]	<0.001

PHQ-4, 4-item Patient Health Questionnaire; MVPA, moderate to vigorous physical activity; TyG, triglyceride-glucose.

^a^ The Townsend deprivation index is a composite measure of socioeconomic deprivation.

^b^ Divided into two categories: college or university degree, and others (including A levels/AS levels or equivalent, O levels/GCSEs or equivalent, CSEs or equivalent, NVQ or HND or HNC or equivalent, other professional qualifications).

^c^ Quantified as average grams per day based on consumption frequency and weekly or monthly volumes of six beverage types.

^d^ Categorized according to the guideline-recommended threshold.

^e^ Assessed based on dietary recommendations for cardiovascular health, with adherence to at least half of the 10 recommended food components defined as a healthy diet.

### Independent association of TyG index or PHQ-4 with incident CVD and MI

During a median follow-up of 13.6 years, 22,867 incident CVD cases and 7,649 incident MI cases were recorded (**Supplementary Table 3**). The incidence rates of CVD across TyG index tertiles 1 to 3 were 4.68 (95% CI: 4.55–4.81), 6.94 (95% CI: 6.78–7.10), and 9.56 (95% CI: 9.38–9.75) per 1,000 person-years, respectively. Corresponding incidence rates of MI were 1.30 (95% CI: 1.24–1.37), 2.24 (95% CI: 2.15–2.33), and 3.38 (95% CI: 3.27–3.49) per 1,000 person-years. Each standard deviation (SD) increase in the TyG index was associated with a 15% higher risk of incident CVD (HR: 1.15; 95% CI: 1.13–1.16) and a 24% higher risk of incident MI (HR: 1.24; 95% CI: 1.22–1.27) (**Supplementary Table 3**). Compared with participants in TyG tertile 1, those in higher tertiles had significantly elevated risks of incident CVD and MI (**Supplementary Table 3**). After multivariable adjustment, the hazard ratios (95% CIs) for CVD were 1.15 (1.11–1.19) for tertile 2 and 1.36 (1.32–1.41) for tertile 3 (*P* for trend < 0.001). Similarly, the HRs (95% CIs) for MI were 1.33 (1.24–1.42) for tertile 2 and 1.68 (1.58–1.79) for tertile 3 (*P* for trend < 0.001). The RCS regression model revealed positive nonlinear dose-response relationships of TyG index with incident CVD and MI (all overall *P* < 0.001, all nonlinear *P* < 0.001) (**Supplementary Figure 2**).

The incidence rates of CVD across PHQ-4 scores of 0, 1, and ≥ 2 were 6.84 (95% CI: 6.70–6.98), 6.95 (95% CI: 6.77–7.14), and 7.39 (95% CI: 7.23–7.55) per 1,000 person-years, respectively. The corresponding incidence rates of MI were 2.28 (95% CI: 2.20–2.37), 2.21 (95% CI: 2.11–2.31), and 2.41 (95% CI: 2.32–2.51) per 1,000 person-years (**Table 2**). After multivariable adjustment, each one-point increase in the PHQ-4 score was associated with a 7% higher risk of incident CVD (HR: 1.07; 95% CI: 1.07–1.08) and a 7% higher risk of incident MI (HR: 1.07; 95% CI: 1.06–1.08) (**Table 2**). Compared with participants with PHQ-4 score = 0, those with higher PHQ-4 scores demonstrated significantly increased risks of incident CVD and MI (**Table 2**). After multivariable adjustment, the HRs (95% CIs) for CVD were 1.18 (1.14–1.22) for PHQ-4 score = 1 and 1.35 (1.31–1.39) for PHQ-4 score ≥ 2 (*P* for trend <0.001). Similarly, the HRs (95% CIs) for incident MI were 1.12 (1.06–1.19) for PHQ-4 score = 1 and 1.30 (1.23–1.37) for PHQ-4 score ≥ 2 (*P* for trend <0.001). Inverse L-shaped associations between PHQ-4 scores and the risks of incident CVD and MI were observed, with the risk notably increasing when the PHQ-4 score reached approximately 1 (**Supplementary Figure 3**).

**Table 2:** Associations of PHQ-4 with incident CVD and MI and mediation proportion attributed to TyG.

Variables	Incidence/ Person years	Incidence rate per 1000 person years (95% CI)	Age and sex adjusted hazard ratio (95% CI)	Multivariate adjusted hazard ratio^a^ (95% CI)	Mediation proportion^b^ (95% CI)
Incident CVD					
PHQ-4 (per1-score)	22867/3241521	7.05 (6.96-7.15)	1.10 (1.09-1.11)	1.07 (1.07-1.08)	0.053 (0.045-0.061)
PHQ-4					
0	9180/1341482	6.84 (6.70-6.98)	1.00 (reference)	1.00 (reference)	–
1	5602/805473	6.95 (6.77-7.14)	1.21 (1.17-1.25)	1.18 (1.14-1.22)	0.028 (-0.016-0.142)
≥2	8085/1094566	7.39 (7.23-7.55)	1.47 (1.43-1.52)	1.35 (1.31-1.39)	0.053 (0.045-0.063)
*P* for trend			<0.001	<0.001	
Incident MI					
PHQ-4 (per1-score)	7649/3311466	2.31 (2.26-2.36)	1.10 (1.09-1.11)	1.07 (1.06-1.08)	0.091 (0.076-0.110)
PHQ-4					
0	3125/1368133	2.28 (2.20-2.37)	1.00 (reference)	1.00 (reference)	–
1	1819/822756	2.21 (2.11-2.31)	1.16 (1.10-1.23)	1.12 (1.06-1.19)	1.480 (-0.727-0.850)^c^
≥2	2705/1120578	2.41 (2.32-2.51)	1.42 (1.35-1.50)	1.30 (1.23-1.37)	0.093 (0.074-0.125)
*P* for trend			<0.001	<0.001	

TyG, triglyceride-glucose; PHQ-4, 4-item Patient Health Questionnaire; CVD, cardiovascular disease; MI, myocardial infarction.

^a^ Multivariate adjusted hazard ratios were adjusted for age, sex, race, educational level, employment status, Townsend Deprivation Index, smoking status, alcohol consumption, physical activity, diet, and self-reported medication use (including antihypertensive drugs, lipid-lowering drugs, or insulin).

^b^ In the mediation analysis, PHQ-4 = 0 was set as the reference group, and the mediation proportion was statistically significant when its confidence interval did not include 0.

^c^ For the PHQ-4 = 1 vs 0 stratum, the proportion mediated exceeds 1.0 due to inconsistent mediation (the direct and indirect effects have opposite signs).

### Mediation analysis of the TyG index on associations of PHQ-4 with incident CVD and MI

The TyG index mediated 5.3% of the association between continuous PHQ-4 scores and incident CVD, and 9.1% of the association with incident MI (**Table 2**). When comparing participants with a PHQ-4 score of 1 to those with a score of 0, the proportion mediated by the TyG index was not statistically significant (**Table 2**). However, when comparing PHQ-4 ≥ 2 group to PHQ-4 = 0 group, the TyG index mediated 5.3% (95% CI: 4.5%–6.3%) of the association with incident CVD and 9.3% (95% CI: 7.4%–12.5%) with incident MI (**Table 2**). The results remained consistent after we excluded participants who had incident CVD or MI within the first two years of follow-up, additionally adjusting for the quadratic terms of age in model, and using multiple imputation to impute all missing independent variables and covariates (**Supplementary Tables 4**–**6**). Sensitivity analyses using alternative PHQ-4 thresholds demonstrated that the TyG index significantly mediated 3.4% of the association between PHQ-4 scores = 1–2 (vs. 0) and incident CVD, 5.5% of the association between PHQ-4 scores ≥ 3 (vs. 0) and incident CVD, as well as 9.2% of the association between PHQ-4 scores ≥ 3 (vs. 0) and incident MI (**Supplementary Table 7**).

### Interaction between TyG index and PHQ-4 status on incident CVD and MI

**Figure 1:**
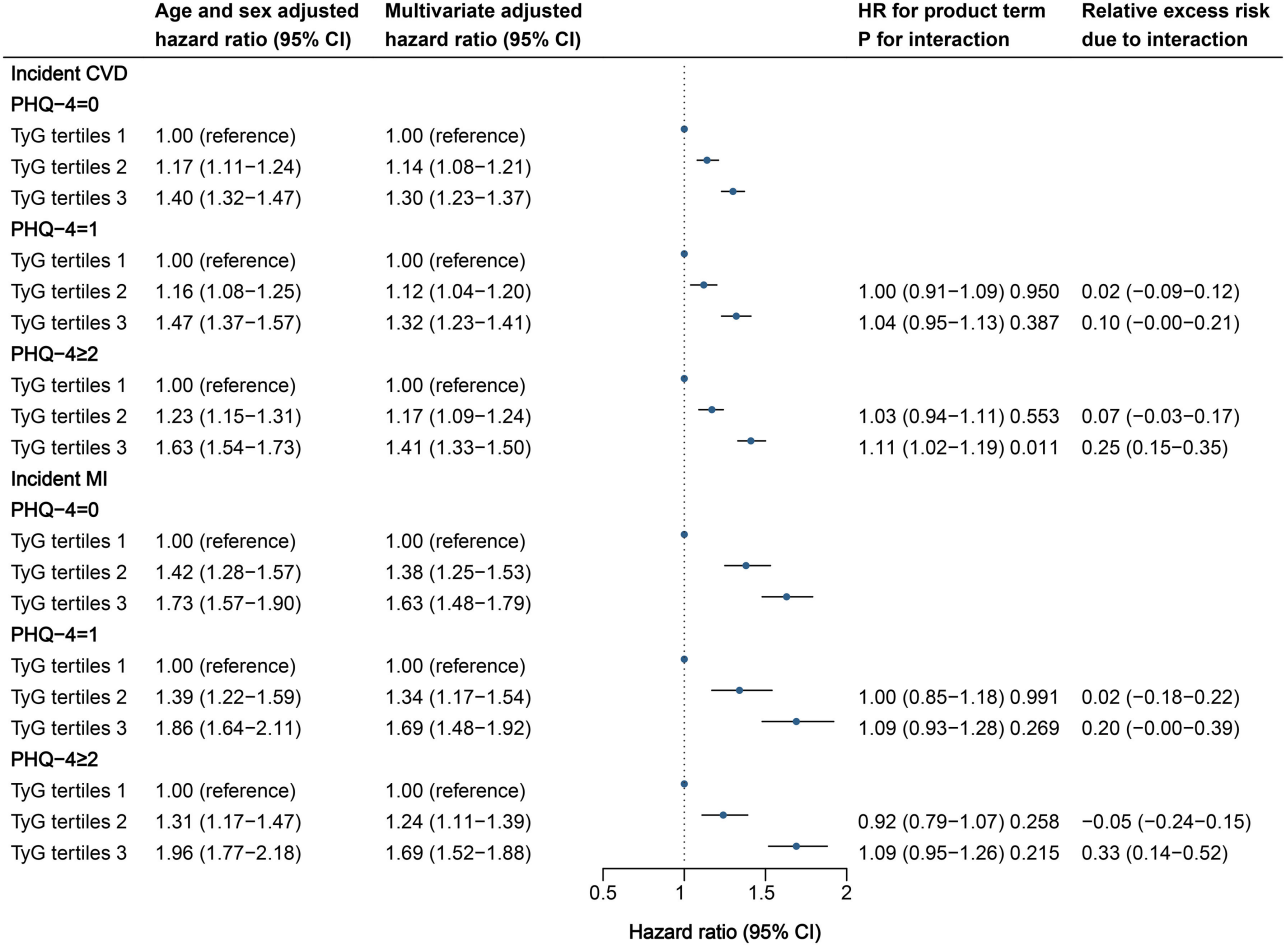
Associations of TyG index with incident CVD and MI by PHQ-4 status. TyG, triglyceride-glucose; PHQ-4, 4-item Patient Health Questionnaire; CVD, cardiovascular disease; MI, myocardial infarction. Multivariate adjusted hazard ratios were adjusted for age, sex, race, educational level, employment status, Townsend Deprivation Index, smoking status, alcohol consumption, physical activity, diet, and self-reported medication use (including antihypertensive drugs, lipid-lowering drugs, or insulin). Multiplicative interaction was evaluated using hazard ratios for the product term between TyG (tertiles) and PHQ-4 status (0, 1, and ≥2), and the multiplicative interaction was statistically significant when its confidence interval did not include 1. Additive interaction was evaluated using relative excess risk due to interaction between TyG (tertiles) and PHQ-4 status (0, 1, and ≥2), and the additive interaction was statistically significant when its confidence interval did not include 0.

A higher TyG index was associated with increased risks of incident CVD and MI across all PHQ-4 status groups, with the associations being stronger among participants with a PHQ-4 score ≥ 2 (**Figure 1**). For example, the HRs (95%CIs) for those with TyG tertile 3 compared with TyG tertile 1 for incident CVD were 1.30 (1.23–1.37) in the PHQ-4 = 0 group, 1.32 (1.23–1.41) in the PHQ-4 = 1 group, and 1.41 (1.33–1.50) in the PHQ-4 ≥ 2 group. Both multiplicative (HR for interaction: 1.11; 95%CI: 1.02–1.19) and additive interactions (RERI: 0.25; 95%CI: 0.15–0.35) were observed between TyG tertile 3 and a PHQ-4 score ≥ 2 on incident CVD. For MI, the HRs (95%CIs) for those with TyG tertile 3 compared with TyG tertile 1 were 1.63 (1.48–1.79) in PHQ-4 = 0 group, 1.69 (1.48–1.92) in PHQ-4 = 1 group, and 1.69 (1.52–1.88) in PHQ-4 ≥ 2 group. Additive interactions (RERI: 0.33; 95%CI: 0.14–0.52) were observed between TyG tertile 3 and a PHQ-4 score ≥ 2 on incident MI. Results were not materially changed in all sensitivity analyses (**Supplementary Tables 8**–**10**). Repeating the analyses using alternative thresholds for defining PHQ-4 status, additive interactions were observed between TyG tertile 3 and PHQ-4 scores = 1–2 or ≥ 3 on incident CVD and MI (**Supplementary Table 11**).

### Joint associations of TyG index and PHQ-4 status with incident CVD and MI

**Figure 2:**
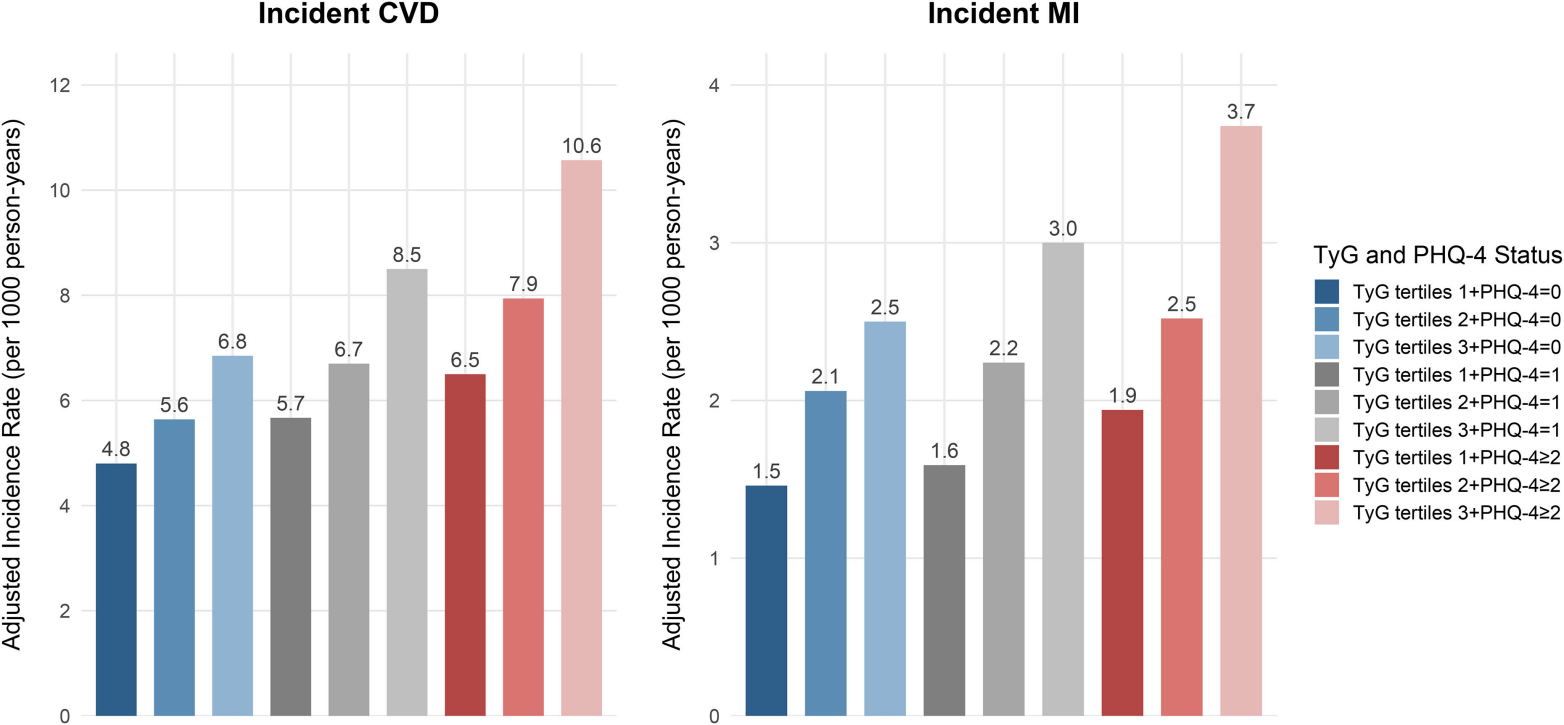
Age- and sex-adjusted incidence rates (per 1,000 person-years) of incident CVD and MI across groups of TyG and PHQ-4 status. TyG, triglyceride-glucose; PHQ-4, 4-item Patient Health Questionnaire; CVD, cardiovascular disease; MI, myocardial infarction.

**Figure 3:**
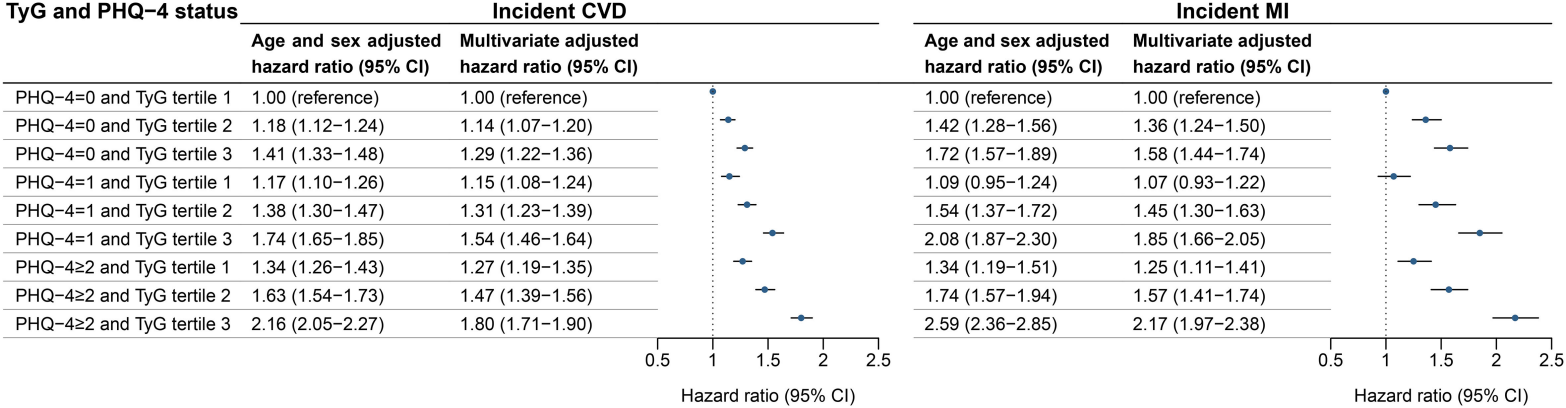
Joint associations of TyG index and PHQ-4 status with incident CVD and MI. TyG, triglyceride-glucose; PHQ-4, 4-item Patient Health Questionnaire; CVD, cardiovascular disease; MI, myocardial infarction. Multivariate adjusted hazard ratios were adjusted for age, sex, race, educational level, employment status, Townsend Deprivation Index, smoking status, alcohol consumption, physical activity, diet, and self-reported medication use (including antihypertensive drugs, lipid-lowering drugs, or insulin).

Age- and sex-adjusted incidence rates (per 1,000 person-years) for CVD and MI were higher among individuals with elevated TyG index and PHQ-4 scores (**Figure 2**). The lowest rates occurred in participants with PHQ-4 = 0 and TyG tertile 1 (CVD: 4.8; MI: 1.5), while the highest rates were observed in those with PHQ-4 ≥ 2 and TyG tertile 3 (CVD: 10.6; MI: 3.7). Compared with the reference group (PHQ-4 = 0 and TyG tertile 1), all other exposure combinations demonstrated significantly elevated CVD risk (**Figure 3**). The greatest synergistic risk was observed in individuals with PHQ-4 ≥ 2 and TyG tertile 3 (HR: 1.80; 95%CI: 1.71–1.90). For MI, nearly all combined groups were also associated with a higher risk, except for participants with PHQ-4 = 1 and TyG tertile 1, in whom the association was not statistically significant (HR: 1.07; 95% CI: 0.93–1.22). The highest risk of incident MI was similarly observed in those with a PHQ-4 score ≥2 and TyG tertile 3 (HR: 2.17; 95% CI: 1.97–2.38). The results of all sensitivity analyses were consistent with the main analyses (**Supplementary Figures 4**–**6**). When PHQ-4 scores were categorized as 0, 1–2, and ≥ 3, the greatest synergistic risks of incident CVD (HR: 1.92; 95% CI: 1.81–2.03) and MI (HR: 2.33; 95% CI: 2.10–2.58) were observed among individuals with PHQ-4 score ≥ 3 and TyG tertile 3 (**Supplementary Figure 7**).

### Subgroup analysis

The TyG index significantly mediated the association of PHQ-4 scores with the risks of incident CVD and MI across all subgroups stratified by age (< 65 and ≥ 65 years), sex (male and female), and race (white people and non-white people) (**Supplementary Tables 12**–**14**). Notably, the proportion of mediation attributed to the TyG index was greater in younger than in older adults, and in females than in males (**Supplementary Tables 12**, **13**).

Elevated TyG index was consistently associated with increased risks of incident CVD and MI across all PHQ-4 categories, regardless of age, sex, or race (**Supplementary Table 15**-**20**). Notably, among younger adults, both males and females, and white participants, significant interactions were observed between TyG tertiles 3 and PHQ-4 ≥ 2 on incident CVD (**Supplementary Tables 15**, **17**, **19**).

The joint associations of the TyG index and PHQ-4 status with incident CVD and MI were stronger in younger adults and females, with significant interactions observed in the PHQ-4 ≥ 2 and TyG tertile 3 subgroup (all P for interaction < 0.05) (**Supplementary Tables 21**–**23**).

## Discussion

In this large cohort study, clear mental health symptoms (PHQ-4 score ≥ 2) were associated with higher risks of incident CVD and MI. IR reflected by the TyG index mediated 5.3% and 9.3% of the association of clear mental health symptoms with incident CVD and MI, respectively, whereas no significant mediation effect was observed for mild symptoms (PHQ-4 score = 1). Synergistic interactions were observed between clear mental health symptoms and pronounced IR (TyG tertile 3) on the risks of incident CVD and MI, with the highest risks observed among these participants.

Although previous studies have shown that either depression or anxiety is associated with an increased risk of CVD (32–36), the use of the PHQ-4 can capture the combined burden of these frequently co-occurring mental health conditions. Our study revealed that the relationship of the PHQ-4 score with incident CVD followed an inverse L-shaped association, with the risk rising notably when scores exceeded approximately 1 (mild mental health symptoms). We further found that individuals with mild to clear mental health symptoms had significantly elevated CVD risk, underscoring the importance of mental health management in CVD prevention.

Studies have shown that mental health symptoms often coexist with IR (8), and both factors may promote CVD through shared mechanisms such as chronic inflammation, abnormalities in insulin signaling, dysregulation of the hypothalamic-pituitary-adrenal axis, and intestinal dysbiosis (37, 38). Additionally, mental health symptoms may contribute to the development of IR, potentially through immune dysregulation (39, 40) and behavioral changes, including smoking, physical inactivity, poor diet quality, and overweight or obesity, thereby further increasing CVD risk (32, 36, 41). However, the role of IR in the pathway linking mental health symptoms to incident CVD remains unclear.

Our findings further demonstrated that the TyG index mediated 5.3% and 9.3% of the associations between clear mental health symptoms and incident CVD and MI, respectively, whereas no significant mediation effect was observed for mild symptoms. Previous studies have reported that individual metabolic factors may mediate the relationship between single mental health conditions and incident CVD (17–19). For instance, a cross-sectional study identified metabolic syndrome as a linking pathway between psychological distress and CVD risk (17). Similarly, a prospective Australian study found that obesity metrics mediated 7.7%–10.4% of the association between depression and CVD (18). More recently, a UK Biobank study showed that single metabolic factors, such as systolic blood pressure, hemoglobin A1c, or low-density lipoprotein cholesterol, mediated 4.3%–10.4% of the association between depression or anxiety and CVD (19). However, these investigations examined isolated mediators rather than integrated metabolic pathways. Moreover, since most prior investigations have focused on depression or anxiety individually, and given that these conditions frequently coexist, assessing the overall mental health burden may provide a more comprehensive representation of the real-world impact on cardiovascular outcomes.

The observed mediating role of IR, as reflected by the TyG index, suggests that IR may represent a partial and modest biological pathway linking clear mental health symptoms to CVD. The modest mediation proportion indicated that reducing cardiovascular risk could not be achieved through mitigating IR alone, and other measures to tackle mental health symptoms are still needed. By contrast, the absence of a significant mediation effect for PHQ-4 score = 1 implies that alleviating mild mental health symptoms may require strategies beyond targeting IR, and alternative interventions addressing emotional distress are needed.

In our study, synergistic effects were observed between clear mental health symptoms (PHQ-4 score ≥ 2) and pronounced IR (TyG tertile 3) on the risk of incident CVD, with the highest risk observed among participants with both conditions. To confirm the specific threshold at which mental health symptoms interact with IR, in sensitivity analyses using alternative PHQ-4 thresholds, significant interactions were also detected between TyG tertile 3 and PHQ-4 scores of 1–2 or ≥ 3. Therefore, among individuals with PHQ-4 score ≥ 2, the detrimental associations of elevated TyG index with health outcomes were stronger. These findings underscore the necessity of mitigating IR, especially among those with clear mental health symptoms (PHQ-4 score ≥ 2) who are more vulnerable to IR. The TyG index might serve as an adjunctive tool for risk stratification that could be integrated into metabolic monitoring for this vulnerable subgroup. Evidence suggests that IR may also increase vulnerability to mental health disorders through mechanisms involving chronic low-grade inflammation, neurotransmitter imbalances, and defective brain insulin signaling (42, 43). This cyclical relationship between mental health symptoms and IR may further intensify the risk of CVD. Collectively, mental health symptoms and IR mutually influence and exacerbate each other, underscoring the need for integrated interventions targeting both metabolic factors and mental health to reduce CVD burden.

In subgroup analyses, the mediation effect of IR and its joint associations with mental health symptoms on incident CVD were stronger in females and younger adults. Sex-related differences in neuroendocrine responses to psychosocial stress may make women with mental health symptoms more susceptible to IR (44). Moreover, estrogen dynamics in modulating adiponectin and insulin sensitivity may explain heightened metabolic vulnerability, making the IR pathway account for a relatively larger share of cardiovascular risk (45). Compared with young populations, the elderly are more likely to have multiple comorbidities and be exposed to more cardiovascular risk factors, which to some extent attenuated the joint contribution of mental health symptoms and IR to CVD. The amplified joint risk when mental health symptoms co-occur with IR supports integrating mental health assessment with metabolic risk management, offering greater marginal benefits for dual-target prevention, particularly in women and younger adults. However, these hypotheses require further validation in future studies.

This study has several limitations. First, information on mental health symptoms was self-reported and therefore likely prone to recall or social desirability bias. Second, we could not capture the long-term mental health symptoms trajectories and TyG index changes, so the observed associations might be attenuated due to nondifferential misclassification bias. Future studies with repeated measurements are preferred. Third, the participants were mostly white people, which limits the generalizability of our findings to other populations. The number of participants and events might be insufficient among the non-white subgroup and the results should be cautiously interpreted. Fourth, the large number of participants excluded due to missing data may introduce selection bias. Nevertheless, we conducted sensitivity analyses using multiple imputation for missing data, and the results remained consistent with our primary findings, demonstrating the robustness of our conclusions. Fifth, although we performed sensitivity analyses by excluding early incident events to reduce potential reverse causation, the potential remains that preclinical CVD or subclinical metabolic disease might have subtly influenced mental health status at baseline. Finally, given the nature of observational studies, residual confounding was still possible and causal inference cannot be made.

## Conclusions

In a large-scale prospective UK cohort, clear mental health symptoms (PHQ-4 score ≥ 2) were significantly associated with higher incident CVD risk, with IR reflected by TyG index partially and modestly mediating these associations. Targeting IR may attenuate the adverse cardiovascular risk associated with clear mental health symptoms. Furthermore, the synergistic effect between PHQ-4 score ≥ 2 and pronounced IR (the highest TyG tertile) on incident CVD highlights the importance of comprehensive interventions to reduce CVD burden, particularly among individuals with clear mental health symptoms who are more vulnerable to IR.

## Data Availability

Publicly available datasets were analyzed in this study. This data can be found here: https://www.ukbiobank.ac.uk/.
