# Supplementary material for: Associations of mental health symptoms and triglyceride-glucose index with incident cardiovascular disease: a cohort study from the UK Biobank
Source: Front Endocrinol (Lausanne). 2026 Jun 24;17:1874050. doi: 10.3389/fendo.2026.1874050 (PMC13341541; doi:10.3389/fendo.2026.1874050)
Supplement: Supplementary file 1 [file DataSheet1.docx]

**Supplementary material**

**Supplementary Table 1.** Baseline characteristics of the study population according to TyG tertile

**Supplementary Table 2.** Baseline characteristics of participants included or excluded from the current study

**Supplementary Table 3.** Independent association of TyG with incident CVD and MI

**Supplementary Table 4.** Associations of PHQ-4 with incident CVD and MI and mediation proportion attributed to TyG after excluding the cases that occurred within first 2 years of follow-up

**Supplementary Table 5.** Associations of PHQ-4 with incident CVD and MI and mediation proportion attributed to TyG after further inclusion of quadratic terms of age in the model

**Supplementary Table 6.** Associations of PHQ-4 with incident CVD and MI and mediation proportion attributed to TyG after using multiple imputations with chained equations

**Supplementary Table 7.** Associations of PHQ-4 with incident CVD and MI and mediation proportion attributed to TyG when using additional threshold for defining PHQ-4 status

**Supplementary Table 8.** Associations of TyG index with incident CVD and MI by PHQ-4 status after excluding the cases that occurred within first 2 years of follow-up

**Supplementary Table 9.** Associations of TyG index with incident CVD and MI by PHQ-4 status after further inclusion of quadratic terms of age in the model

**Supplementary Table 10.** Associations of TyG index with incident CVD and MI by PHQ-4 status after using multiple imputations with chained equations

**Supplementary Table 11.** Associations of TyG index with incident CVD and MI by PHQ-4 status when using additional threshold for defining PHQ-4 status

**Supplementary Table 12.** Associations of PHQ-4 with incident CVD and MI and mediation proportion attributed to TyG stratified by age

**Supplementary Table 13.** Associations of PHQ-4 with incident CVD and MI and mediation proportion attributed to TyG stratified by sex

**Supplementary Table 14.** Associations of PHQ-4 with incident CVD and MI and mediation proportion attributed to TyG stratified by race

**Supplementary Table 15.** Associations of TyG index with incident CVD by PHQ-4 status stratified by age

**Supplementary Table 16.** Associations of TyG index with incident MI by PHQ-4 status stratified by age

**Supplementary Table 17.** Associations of TyG index with incident CVD by PHQ-4 status stratified by sex

**Supplementary Table 18.** Associations of TyG index with incident MI by PHQ-4 status stratified by sex

**Supplementary Table 19.** Associations of TyG index with incident CVD by PHQ-4 status stratified by race

**Supplementary Table 20.** Associations of TyG index with incident MI by PHQ-4 status stratified by race

**Supplementary Table 21.** Joint associations of TyG index and PHQ-4 status with incident CVD and MI stratified by age

**Supplementary Table 22.** Joint associations of TyG index and PHQ-4 status with incident CVD and MI stratified by sex

**Supplementary Table 23.** Joint associations of TyG index and PHQ-4 status with incident CVD and MI stratified by race

**Supplementary Figure 1.** Flowchart of participants included in the analysis

**Supplementary Figure 2.** Dose-response associations of TyG index with incident CVD (A) and MI (B)

**Supplementary Figure 3.** Dose-response associations of PHQ-4 scores with incident CVD (A) and MI (B)

**Supplementary Figure 4.** Joint associations of TyG index and PHQ-4 status with incident CVD and MI after excluding the cases that occurred within first 2 years of follow-up

**Supplementary Figure 5.** Joint associations of TyG index and PHQ-4 status with incident CVD and MI after further inclusion of quadratic terms of age in the model

**Supplementary Figure 6.** Joint associations of TyG index and PHQ-4 status with incident CVD and MI after using multiple imputations with chained equations

**Supplementary Figure 7.** Joint associations of TyG index and PHQ-4 status with incident CVD and MI when using additional threshold for defining PHQ-4 status

This material has been provided by the authors to give readers additional information about their work.

**Supplementary Table 1. Baseline characteristics of the study population according to TyG tertiles**

|  | **TyG tertile 1 (N=82736)** | **TyG tertile 2 (N=82736)** | **TyG tertile 3 (N=85244)** | ***P* value** |
| --- | --- | --- | --- | --- |
| Age, years | 54.4 (8.3) | 56.9 (7.9) | 57.3 (7.7) | <0.001 |
| Sex, n (%) |  |  |  | <0.001 |
| Female | 54214 (65.5%) | 44602 (53.9%) | 34974 (41.0%) |  |
| Male | 28522 (34.5%) | 38134 (46.1%) | 50270 (59.0%) |  |
| Race, n (%) |  |  |  | <0.001 |
| Asian | 963 (1.2%) | 1138 (1.4%) | 1499 (1.8%) |  |
| Black | 1630 (2.0%) | 694 (0.8%) | 404 (0.5%) |  |
| Other | 1096 (1.3%) | 820 (1.0%) | 855 (1.0%) |  |
| White | 79047 (95.5%) | 80084 (96.8%) | 82486 (96.8%) |  |
| Townsend deprivation index, n (%) | | | | <0.001 |
| Least deprived | 18620 (22.5%) | 18655 (22.5%) | 18384 (21.6%) |  |
| Intermediate deprived | 50770 (61.4%) | 50987 (61.6%) | 52524 (61.6%) |  |
| Most deprived | 13346 (16.1%) | 13094 (15.8%) | 14336 (16.8%) |  |
| Educational level, n (%) |  |  |  | <0.001 |
| Others | 36711 (44.4%) | 40605 (49.1%) | 44130 (51.8%) |  |
| College or university degree | 46025 (55.6%) | 42131 (50.9%) | 41114 (48.2%) |  |
| Employment status, n (%) |  |  |  | <0.001 |
| Employed | 55598 (67.2%) | 48098 (58.1%) | 47902 (56.2%) |  |
| Retired/unemployed | 27138 (32.8%) | 34638 (41.9%) | 37342 (43.8%) |  |
| Self-reported use of antihypertensive drugs, n (%) | | | | <0.001 |
| No | 73956 (89.4%) | 68844 (83.2%) | 65307 (76.6%) |  |
| Yes | 8780 (10.6%) | 13892 (16.8%) | 19937 (23.4%) |  |
| Self-reported use of lipid-lowering drugs, n (%) | | | | <0.001 |
| No | 76231 (92.1%) | 72484 (87.6%) | 69196 (81.2%) |  |
| Yes | 6505 (7.9%) | 10252 (12.4%) | 16048 (18.8%) |  |
| Self-reported use of insulin, n (%) | | | | <0.001 |
| No | 82318 (99.5%) | 82330 (99.5%) | 83909 (98.4%) |  |
| Yes | 418 (0.5%) | 406 (0.5%) | 1335 (1.6%) |  |
| Smoking status, n (%) |  |  |  | <0.001 |
| Never | 51226 (61.9%) | 47840 (57.8%) | 45111 (52.9%) |  |
| Previous | 25857 (31.3%) | 28314 (34.2%) | 31584 (37.1%) |  |
| Current | 5653 (6.8%) | 6582 (8.0%) | 8549 (10.0%) |  |
| Alcohol intake, g/day | 14.5 (15.0) | 15.2 (16.2) | 17.0 (19.0) | <0.001 |
| Physical activity, n (%) |  |  |  | <0.001 |
| <150 min/wk MVPA | 26424 (31.9%) | 29395 (35.5%) | 34274 (40.2%) |  |
| ≥150 min/wk MVPA | 56312 (68.1%) | 53341 (64.5%) | 50970 (59.8%) |  |
| Diet |  |  |  | <0.001 |
| <5 health diet | 67030 (81.0%) | 68664 (83.0%) | 72537 (85.1%) |  |
| ≥5 health diet | 15706 (19.0%) | 14072 (17.0%) | 12707 (14.9%) |  |
| PHQ-4 | 1.4 (1.9) | 1.4 (1.9) | 1.6 (2.1) | <0.001 |

TyG, triglyceride-glucose; MVPA, moderate to vigorous physical activity; PHQ-4, 4-item Patient Health Questionnaire

^a^ The Townsend deprivation index is a composite measure of socioeconomic deprivation. ^b^ Divided into two categories: college or university degree, and others (including A levels/AS levels or equivalent, O levels/GCSEs or equivalent, CSEs or equivalent, NVQ or HND or HNC or equivalent, other professional qualifications). ^c^ Quantified as average grams per day based on consumption frequency and weekly or monthly volumes of six beverage types. ^d^ Categorized according to the guideline-recommended threshold. ^e^ Assessed based on dietary recommendations for cardiovascular health, with adherence to at least half of the 10 recommended food components defined as a healthy diet.

**Supplementary Table 2.** **Baseline characteristics of participants included or excluded from the current study**

|  | **Excluded^a^ (N=251640)** | **Included (N=250716)** | ***P* value** |
| --- | --- | --- | --- |
| Age, years | 56.88 (8.11) | 56.18 (8.06) | <0.001 |
| Sex, n (%) |  |  | <0.001 |
| Female | 139504 (55.4) | 133790 (53.4) |  |
| Male | 112136 (44.6) | 116926 (46.6) |  |
| Race, n (%) |  |  | <0.001 |
| Asian | 7851 (3.2) | 3600 (1.4) |  |
| Black | 5330 (2.1) | 2728 (1.1) |  |
| Other | 4737 (1.9) | 2771 (1.1) |  |
| White | 230944 (92.8) | 241617 (96.4) |  |
| Townsend deprivation index, n (%)^b^ | | | <0.001 |
| Least deprived | 44969 (17.9) | 55659 (22.2) |  |
| Intermediate deprived | 146481 (58.4) | 154281 (61.5) |  |
| Most deprived | 59564 (23.7) | 40776 (16.3) |  |
| Educational level, n (%)^c^ |  |  | <0.001 |
| Others | 142761 (57.8) | 121446 (48.4) |  |
| College or university degree | 104237 (42.2) | 129270 (51.6) |  |
| Employment status, n (%) |  |  | <0.001 |
| Employed | 135443 (54.0) | 151598 (60.5) |  |
| Retired/unemployed | 115325 (46.0) | 99118 (39.5) |  |
| Self-reported use of antihypertensive drugs, n (%) | | | <0.001 |
| No | 181666 (74.7) | 208107 (83.0) |  |
| Yes | 61369 (25.3) | 42609 (17.0) |  |
| Self-reported use of lipid-lowering drugs, n (%) | | | <0.001 |
| No | 188968 (77.8) | 217911 (86.9) |  |
| Yes | 54067 (22.2) | 32805 (13.1) |  |
| Self-reported use of insulin, n (%) | | | <0.001 |
| No | 239583 (98.6) | 248557 (99.1) |  |
| Yes | 3452 (1.4) | 2159 (0.9) |  |
| Smoking status, n (%) | | | <0.001 |
| Never | 129270 (52.0) | 144177 (57.5) |  |
| Previous | 87246 (35.1) | 85755 (34.2) |  |
| Current | 32174 (12.9) | 20784 (8.3) |  |
| Alcohol intake, g/day^d^ | 16.02 (20.20) | 15.60 (16.87) | <0.001 |
| Physical activity, n (%)^e^ |  |  | <0.001 |
| <150 min/wk MVPA | 69701 (39.3) | 90093 (35.9) |  |
| ≥150 min/wk MVPA | 107538 (60.7) | 160623 (64.1) |  |
| Diet^f^ |  |  | 0.77 |
| <5 health diet | 111248 (83.1) | 208231 (83.1) |  |
| ≥5 health diet | 22637 (16.9) | 42485 (16.9) |  |
| PHQ-4 | 1.78 (2.28) | 1.47 (1.95) | <0.001 |
| TyG | 8.74 (0.58) | 8.70 (0.56) | <0.001 |

MVPA, moderate to vigorous physical activity; PHQ-4, 4-item Patient Health Questionnaire; TyG, triglyceride-glucose

^a^ Those with missing data on the TyG index and PHQ-4 at baseline, those with missing information on covariates, and those diagnosed with CVD at baseline were excluded. ^b^ The Townsend deprivation index is a composite measure of socioeconomic deprivation. ^c^ Divided into two categories: college or university degree, and others (including A levels/AS levels or equivalent, O levels/GCSEs or equivalent, CSEs or equivalent, NVQ or HND or HNC or equivalent, other professional qualifications). ^d^ Quantified as average grams per day based on consumption frequency and weekly or monthly volumes of six beverage types. ^e^ Categorized according to the guideline-recommended threshold.

^f^ Assessed based on dietary recommendations for cardiovascular health, with adherence to at least half of the 10 recommended food components defined as a healthy diet.

**Supplementary Table 3.** **Independent association of TyG with incident CVD and MI**

|  | **Incidence/**  **Person years** | **Incidence rate per 1000 person years (95% CI)** | **Age and sex adjusted hazard ratio (95% CI)** | **Multivariate adjusted hazard ratio^a^ (95% CI)** |
| --- | --- | --- | --- | --- |
| **Incident CVD** |  |  |  |  |
| TyG (per1-SD^b^) | 22867/3241521 | 7.05 (6.96-7.15) | 1.22 (1.20-1.23) | 1.15 (1.13-1.16) |
| TyG (tertiles) |  |  |  |  |
| Tertiles 1 | 5098/1089903 | 4.68 (4.55-4.81) | 1.00 (reference) | 1.00 (reference) |
| Tertiles 2 | 7425/1069686 | 6.94 (6.78-7.10) | 1.20 (1.16-1.24) | 1.15 (1.11-1.19) |
| Tertiles 3 | 10344/1081932 | 9.56 (9.38-9.75) | 1.52 (1.47-1.58) | 1.36 (1.32-1.41) |
| *P* for trend |  |  | <0.001 | <0.001 |
| **Incident MI** |  |  |  |  |
| TyG (per1-SD^b^) | 7649/3311466 | 2.31 (2.26-2.36) | 1.32 (1.29-1.34) | 1.24 (1.22-1.27) |
| TyG (tertiles) |  |  |  |  |
| Tertiles 1 | 1441/1105945 | 1.30 (1.24-1.37) | 1.00 (reference) | 1.00 (reference) |
| Tertiles 2 | 2448/1092238 | 2.24 (2.15-2.33) | 1.39 (1.30-1.48) | 1.33 (1.24-1.42) |
| Tertiles 3 | 3760/1113283 | 3.38 (3.27-3.49) | 1.88 (1.76-1.99) | 1.68 (1.58-1.79) |
| *P* for trend |  |  | <0.001 | <0.001 |

TyG, triglyceride-glucose; SD, standard deviation; CVD, cardiovascular disease; MI, myocardial infarction

^a^ Multivariate adjusted hazard ratios were adjusted for age, sex, race, educational level, employment status, Townsend Deprivation Index, smoking status, alcohol consumption, physical activity, diet, and self-reported medication use (including antihypertensive drugs, lipid-lowering drugs, or insulin). ^b^ The SD of TyG index is 0.56.

**Supplementary Table 4.** **Associations of PHQ-4 with incident CVD and MI and mediation proportion attributed to TyG after excluding the cases that occurred within first 2 years of follow-up**

|  | **Age and sex adjusted hazard ratio (95% CI)** | **Multivariate adjusted hazard ratio^a^ (95% CI)** | **Mediation proportion^b^**  **(95% CI)** |
| --- | --- | --- | --- |
| **Incident CVD** |  |  |  |
| PHQ-4 (per1-score) | 1.10 (1.09-1.10) | 1.07 (1.06-1.08) | 0.053 (0.045-0.062) |
| PHQ-4 |  |  |  |
| 0 | 1.00 (reference) | 1.00 (reference) | - |
| 1 | 1.20 (1.16-1.25) | 1.17 (1.13-1.21) | 0.025 (-0.022-0.171) |
| ≥2 | 1.45 (1.40-1.49) | 1.33 (1.29-1.38) | **0.053 (0.044-0.064)** |
| *P* for trend | <0.001 | <0.001 |  |
| **Incident MI** |  |  |  |
| PHQ-4 (per1-score) | 1.10 (1.08-1.11) | 1.07 (1.06-1.08) | 0.091 (0.075-0.115) |
| PHQ-4 |  |  |  |
| 0 | 1.00 (reference) | 1.00 (reference) | - |
| 1 | 1.16 (1.09-1.23) | 1.12 (1.05-1.19) | 1.352 (-1.015-0.698)^c^ |
| ≥2 | 1.41 (1.33-1.49) | 1.29 (1.22-1.36) | **0.093 (0.071-0.124)** |
| *P* for trend | <0.001 | <0.001 |  |

TyG, triglyceride-glucose; PHQ-4, 4-item Patient Health Questionnaire; CVD, cardiovascular disease; MI, myocardial infarction

^a^ Multivariate adjusted hazard ratios were adjusted for age, sex, race, educational level, employment status, Townsend Deprivation Index, smoking status, alcohol consumption, physical activity, diet, and self-reported medication use (including antihypertensive drugs, lipid-lowering drugs, or insulin). ^b^ In the mediation analysis, PHQ-4 =0 was set as the reference group, and the mediation proportion was statistically significant when its confidence interval did not include 0. ^c^ For the PHQ-4 = 1 vs 0 stratum, the proportion mediated exceeds 1.0 due to inconsistent mediation (the direct and indirect effects have opposite signs).

**Supplementary Table 5.** **Associations of PHQ-4 with incident CVD and MI and mediation proportion attributed to TyG after further inclusion of** **quadratic terms of age in the model**

|  | **Age and sex adjusted hazard ratio (95% CI)** | **Multivariate adjusted hazard ratio^a^ (95% CI)** | **Mediation proportion^b^**  **(95% CI)** |
| --- | --- | --- | --- |
| **Incident CVD** |  |  |  |
| PHQ-4 (per1-score) | 1.10 (1.09-1.11) | 1.07 (1.07-1.08) | 0.051 (0.045-0.059) |
| PHQ-4 |  |  |  |
| 0 | 1.00 (reference) | 1.00 (reference) | - |
| 1 | 1.21 (1.17-1.25) | 1.18 (1.14-1.22) | 0.036 (-0.010-0.216) |
| ≥2 | 1.47 (1.43-1.52) | 1.35 (1.31-1.39) | **0.052 (0.044-0.061)** |
| *P* for trend | <0.001 | <0.001 |  |
| **Incident MI** |  |  |  |
| PHQ-4 (per1-score) | 1.10 (1.09-1.11) | 1.07 (1.06-1.08) | 0.088 (0.072-0.106) |
| PHQ-4 |  |  |  |
| 0 | 1.00 (reference) | 1.00 (reference) | - |
| 1 | 1.16 (1.09-1.23) | 1.12 (1.06-1.19) | 0.806 (-1.054-0.947) |
| ≥2 | 1.42 (1.35-1.50) | 1.30 (1.23-1.37) | **0.090 (0.072-0.116)** |
| *P* for trend | <0.001 | <0.001 |  |

TyG, triglyceride-glucose; PHQ-4, 4-item Patient Health Questionnaire; CVD, cardiovascular disease; MI, myocardial infarction

^a^ Multivariate adjusted hazard ratios were adjusted for age, age^2^, sex, race, educational level, employment status, Townsend Deprivation Index, smoking status, alcohol consumption, physical activity, diet, and self-reported medication use (including antihypertensive drugs, lipid-lowering drugs, or insulin). ^b^ In the mediation analysis, PHQ-4 =0 was set as the reference group, and the mediation proportion was statistically significant when its confidence interval did not include 0.

**Supplementary Table 6.** **Associations of PHQ-4 with incident CVD and MI and mediation proportion attributed to TyG after using multiple imputations with chained equations**

|  | **Age and sex adjusted hazard ratio (95% CI)** | **Multivariate adjusted hazard ratio^a^ (95% CI)** | **Mediation proportion^b^**  **(95% CI)** |
| --- | --- | --- | --- |
| **Incident CVD** |  |  |  |
| PHQ-4 (per1-score) | 1.10 (1.10-1.11) | 1.07 (1.07-1.08) | 0.048 (0.043-0.054) |
| PHQ-4 |  |  |  |
| 0 | 1.00 (reference) | 1.00 (reference) | - |
| 1 | 1.20 (1.17-1.24) | 1.17 (1.14-1.20) | 0.084 (-0.845-0.568) |
| ≥2 | 1.50 (1.47-1.54) | 1.36 (1.33-1.39) | **0.048 (0.042-0.055)** |
| *P* for trend | <0.001 | <0.001 |  |
| **Incident MI** |  |  |  |
| PHQ-4 (per1-score) | 1.09 (1.09-1.10) | 1.06 (1.05-1.07) | 0.094 (0.080-0.111) |
| PHQ-4 |  |  |  |
| 0 | 1.00 (reference) | 1.00 (reference) | - |
| 1 | 1.15 (1.10-1.21) | 1.12 (1.06-1.17) | -0.478 (-2.293-1.669) |
| ≥2 | 1.44 (1.38-1.50) | 1.29 (1.23-1.34) | **0.092 (0.075-0.116)** |
| *P* for trend | <0.001 | <0.001 |  |

TyG, triglyceride-glucose; PHQ-4, 4-item Patient Health Questionnaire; CVD, cardiovascular disease; MI, myocardial infarction

^a^ Multivariate adjusted hazard ratios were adjusted for age, sex, race, educational level, employment status, Townsend Deprivation Index, smoking status, alcohol consumption, physical activity, diet, and self-reported medication use (including antihypertensive drugs, lipid-lowering drugs, or insulin). ^b^ In the mediation analysis, PHQ-4 =0 was set as the reference group, and the mediation proportion was statistically significant when its confidence interval did not include 0.

**Supplementary Table 7.** **Associations of PHQ-4 with incident CVD and MI and mediation proportion attributed to TyG when using additional threshold for defining PHQ-4 status**

|  | **Age and sex adjusted hazard ratio (95% CI)** | **Multivariate adjusted hazard ratio^a^ (95% CI)** | **Mediation proportion^b^**  **(95% CI)** |
| --- | --- | --- | --- |
| **Incident CVD** |  |  |  |
| PHQ-4 (per1-score) | 1.10 (1.09-1.11) | 1.07 (1.07-1.08) | 0.053 (0.045-0.061) |
| PHQ-4 |  |  |  |
| 0 | 1.00 (reference) | 1.00 (reference) | - |
| 1-2 | 1.23 (1.19-1.26) | 1.19 (1.15-1.22) | **0.034 (0.010-0.093)** |
| ≥3 | 1.61 (1.56-1.67) | 1.44 (1.39-1.49) | **0.055 (0.047-0.065)** |
| *P* for trend | <0.001 | <0.001 |  |
| **Incident MI** |  |  |  |
| PHQ-4 (per1-score) | 1.10 (1.09-1.11) | 1.07 (1.06-1.08) | 0.091 (0.076-0.110) |
| PHQ-4 |  |  |  |
| 0 | 1.00 (reference) | 1.00 (reference) | - |
| 1-2 | 1.17 (1.11-1.23) | 1.13 (1.07-1.19) | 0.368 (-3.168-1.303) |
| ≥3 | 1.57 (1.48-1.66) | 1.39 (1.31-1.48) | **0.092 (0.074-0.117)** |
| *P* for trend | <0.001 | <0.001 |  |

TyG, triglyceride-glucose; PHQ-4, 4-item Patient Health Questionnaire; CVD, cardiovascular disease; MI, myocardial infarction

^a^ Multivariate adjusted hazard ratios were adjusted for age, sex, race, educational level, employment status, Townsend Deprivation Index, smoking status, alcohol consumption, physical activity, diet, and self-reported medication use (including antihypertensive drugs, lipid-lowering drugs, or insulin). ^b^ In the mediation analysis, PHQ-4 =0 was set as the reference group, and the mediation proportion was statistically significant when its confidence interval did not include 0.

**Supplementary Table 8.** **Associations of TyG index with incident CVD and MI by PHQ-4 status** **after excluding the cases that occurred within first 2 years of follow-up**

|  | **Incident CVD** | | | **Incident MI** | | |
| --- | --- | --- | --- | --- | --- | --- |
|  | **Multivariate adjusted hazard ratio^a^ (95% CI)** | **HR for product term,**  ***P* for interaction^b^** | **Relative excess risk due to interaction^c^** | **Multivariate adjusted hazard ratio^a^ (95% CI)** | **HR for product term,**  ***P* for interaction^b^** | **Relative excess risk due to interaction^c^** |
| **PHQ-4=0** |  |  |  |  |  |  |
| TyG tertiles 1 | 1.00 (reference) |  |  | 1.00 (reference) |  |  |
| TyG tertiles 2 | 1.14 (1.08-1.21) |  |  | 1.38 (1.24-1.52) |  |  |
| TyG tertiles 3 | 1.30 (1.23-1.37) |  |  | 1.63 (1.48-1.80) |  |  |
| **PHQ-4=1** |  |  |  |  |  |  |
| TyG tertiles 1 | 1.00 (reference) |  |  | 1.00 (reference) |  |  |
| TyG tertiles 2 | 1.11 (1.03-1.20) | 0.99 (0.90-1.09) 0.838 | 0.01 (-0.10-0.12) | 1.34 (1.17-1.55) | 1.01 (0.85-1.20) 0.944 | 0.03 (-0.17-0.24) |
| TyG tertiles 3 | 1.29 (1.20-1.39) | 1.02 (0.93-1.11) 0.672 | 0.07 (-0.03-0.18) | 1.70 (1.49-1.94) | 1.10 (0.93-1.29) 0.253 | 0.20 (0.00-0.41) |
| **PHQ-4≥2** |  |  |  |  |  |  |
| TyG tertiles 1 | 1.00 (reference) |  |  | 1.00 (reference) |  |  |
| TyG tertiles 2 | 1.17 (1.10-1.25) | 1.03 (0.94-1.12) 0.509 | 0.08 (-0.03-0.18) | 1.24 (1.11-1.40) | 0.92 (0.79-1.08) 0.303 | -0.04 (-0.24-0.16) |
| TyG tertiles 3 | 1.41 (1.32-1.50) | **1.10 (1.02-1.20) 0.017** | **0.24 (0.14-0.34)** | 1.68 (1.50-1.87) | 1.08 (0.94-1.25) 0.286 | **0.31 (0.12-0.51)** |

TyG, triglyceride-glucose; PHQ-4, 4-item Patient Health Questionnaire; CVD, cardiovascular disease; MI, myocardial infarction.

^a^ Multivariate adjusted hazard ratios were adjusted for age, sex, race, educational level, employment status, Townsend Deprivation Index, smoking status, alcohol consumption, physical activity, diet, and self-reported medication use (including antihypertensive drugs, lipid-lowering drugs, or insulin). ^b^ Multiplicative interaction was evaluated using hazard ratios for the product term between TyG (tertiles) and PHQ-4 status (0, 1, and ≥2), and the multiplicative interaction was statistically significant when its confidence interval did not include 1. ^c^ Additive interaction was evaluated using relative excess risk due to interaction between TyG (tertiles) and PHQ-4 status (0, 1, and ≥2), and the additive interaction was statistically significant when its confidence interval did not include 0.

**Supplementary Table 9.** **Associations of TyG index with incident CVD and MI by PHQ-4 status after further inclusion of quadratic terms of age in the model**

|  | **Incident CVD** | | | **Incident MI** | | |
| --- | --- | --- | --- | --- | --- | --- |
|  | **Multivariate adjusted hazard ratio^a^ (95% CI)** | **HR for product term,**  ***P* for interaction^b^** | **Relative excess risk due to interaction^c^** | **Multivariate adjusted hazard ratio^a^ (95% CI)** | **HR for product term,**  ***P* for interaction^b^** | **Relative excess risk due to interaction^c^** |
| **PHQ-4=0** |  |  |  |  |  |  |
| TyG tertiles 1 | 1.00 (reference) |  |  | 1.00 (reference) |  |  |
| TyG tertiles 2 | 1.14 (1.08-1.21) |  |  | 1.38 (1.25-1.52) |  |  |
| TyG tertiles 3 | 1.30 (1.23-1.38) |  |  | 1.63 (1.48-1.79) |  |  |
| **PHQ-4=1** |  |  |  |  |  |  |
| TyG tertiles 1 | 1.00 (reference) |  |  | 1.00 (reference) |  |  |
| TyG tertiles 2 | 1.12 (1.04-1.20) | 1.00 (0.91-1.09) 0.950 | 0.02 (-0.09-0.12) | 1.34 (1.17-1.53) | 1.00 (0.84-1.18) 0.976 | 0.02 (-0.18-0.22) |
| TyG tertiles 3 | 1.32 (1.23-1.42) | 1.04 (0.95-1.13) 0.387 | 0.10 (-0.00-0.21) | 1.68 (1.48-1.91) | 1.09 (0.93-1.28) 0.278 | 0.19 (-0.00-0.39) |
| **PHQ-4≥2** |  |  |  |  |  |  |
| TyG tertiles 1 | 1.00 (reference) |  |  | 1.00 (reference) |  |  |
| TyG tertiles 2 | 1.16 (1.09-1.24) | 1.03 (0.94-1.11) 0.553 | 0.07 (-0.03-0.17) | 1.24 (1.11-1.39) | 0.92 (0.79-1.06) 0.248 | -0.05 (-0.25-0.14) |
| TyG tertiles 3 | 1.41 (1.33-1.49) | **1.11 (1.02-1.19) 0.011** | **0.25 (0.15-0.35)** | 1.68 (1.51-1.87) | 1.09 (0.95-1.25) 0.228 | **0.33 (0.14-0.51)** |

TyG, triglyceride-glucose; PHQ-4, 4-item Patient Health Questionnaire; CVD, cardiovascular disease; MI, myocardial infarction.

^a^ Multivariate adjusted hazard ratios were adjusted for age, age^2^, sex, race, educational level, employment status, Townsend Deprivation Index, smoking status, alcohol consumption, physical activity, diet, and self-reported medication use (including antihypertensive drugs, lipid-lowering drugs, or insulin). ^b^ Multiplicative interaction was evaluated using hazard ratios for the product term between TyG (tertiles) and PHQ-4 status (0, 1, and ≥2), and the multiplicative interaction was statistically significant when its confidence interval did not include 1. ^c^ Additive interaction was evaluated using relative excess risk due to interaction between TyG (tertiles) and PHQ-4 status (0, 1, and ≥2), and the additive interaction was statistically significant when its confidence interval did not include 0.

**Supplementary Table 10.** **Associations of TyG index with incident CVD and MI by PHQ-4 status after using multiple imputations with chained equations**

|  | **Incident CVD** | | | **Incident MI** | | |
| --- | --- | --- | --- | --- | --- | --- |
|  | **Multivariate adjusted hazard ratio^a^ (95% CI)** | **HR for product term,**  ***P* for interaction^b^** | **Relative excess risk due to interaction^c^** | **Multivariate adjusted hazard ratio^a^ (95% CI)** | **HR for product term,**  ***P* for interaction^b^** | **Relative excess risk due to interaction^c^** |
| **PHQ-4=0** |  |  |  |  |  |  |
| TyG tertiles 1 | 1.00 (reference) |  |  | 1.00 (reference) |  |  |
| TyG tertiles 2 | 1.11 (1.06-1.16) |  |  | 1.32 (1.22-1.44) |  |  |
| TyG tertiles 3 | 1.27 (1.22-1.33) |  |  | 1.64 (1.52-1.78) |  |  |
| **PHQ-4=1** |  |  |  |  |  |  |
| TyG tertiles 1 | 1.00 (reference) |  |  | 1.00 (reference) |  |  |
| TyG tertiles 2 | 1.12 (1.06-1.19) | 1.03 (0.96-1.11) 0.436 | 0.05 (-0.03-0.14) | 1.32 (1.18-1.48) | 1.02 (0.89-1.17) 0.806 | 0.05 (-0.12-0.21) |
| TyG tertiles 3 | 1.28 (1.22-1.36) | 1.03 (0.96-1.11) 0.419 | 0.08 (-0.01-0.16) | 1.69 (1.52-1.88) | 1.07 (0.94-1.22) 0.318 | 0.16 (-0.01-0.32) |
| **PHQ-4≥2** |  |  |  |  |  |  |
| TyG tertiles 1 | 1.00 (reference) |  |  | 1.00 (reference) |  |  |
| TyG tertiles 2 | 1.15 (1.09-1.21) | 1.04 (0.97-1.11) 0.267 | 0.08 (0.00-0.16) | 1.19 (1.09-1.31) | 0.91 (0.81-1.03) 0.132 | -0.07 (-0.22-0.09) |
| TyG tertiles 3 | 1.40 (1.33-1.46) | **1.11 (1.04-1.18) <0.001** | **0.25 (0.17-0.33)** | 1.67 (1.54-1.82) | 1.05 (0.94-1.18) 0.371 | **0.27 (0.12-0.42)** |

TyG, triglyceride-glucose; PHQ-4, 4-item Patient Health Questionnaire; CVD, cardiovascular disease; MI, myocardial infarction.

^a^ Multivariate adjusted hazard ratios were adjusted for age, sex, race, educational level, employment status, Townsend Deprivation Index, smoking status, alcohol consumption, physical activity, diet, and self-reported medication use (including antihypertensive drugs, lipid-lowering drugs, or insulin). ^b^ Multiplicative interaction was evaluated using hazard ratios for the product term between TyG (tertiles) and PHQ-4 status (0, 1, and ≥2), and the multiplicative interaction was statistically significant when its confidence interval did not include 1. ^c^ Additive interaction was evaluated using relative excess risk due to interaction between TyG (tertiles) and PHQ-4 status (0, 1, and ≥2), and the additive interaction was statistically significant when its confidence interval did not include 0.

**Supplementary Table 11.** **Associations of TyG index with incident CVD and MI by PHQ-4 status when using additional threshold for defining PHQ-4 status**

|  | **Incident CVD** | | | **Incident MI** | | |
| --- | --- | --- | --- | --- | --- | --- |
|  | **Multivariate adjusted hazard ratio^a^ (95% CI)** | **HR for product term,**  ***P* for interaction^b^** | **Relative excess risk due to interaction^c^** | **Multivariate adjusted hazard ratio^a^ (95% CI)** | **HR for product term,**  ***P* for interaction^b^** | **Relative excess risk due to interaction^c^** |
| **PHQ-4=0** |  |  |  |  |  |  |
| TyG tertiles 1 | 1.00 (reference) |  |  | 1.00 (reference) |  |  |
| TyG tertiles 2 | 1.14 (1.08-1.21) |  |  | 1.38 (1.25-1.53) |  |  |
| TyG tertiles 3 | 1.30 (1.23-1.37) |  |  | 1.63 (1.48-1.79) |  |  |
| **PHQ-4=1 or 2** |  |  |  |  |  |  |
| TyG tertiles 1 | 1.00 (reference) |  |  | 1.00 (reference) |  |  |
| TyG tertiles 2 | 1.15 (1.09-1.22) | 1.03 (0.95-1.11) 0.547 | 0.05 (-0.04-0.14) | 1.37 (1.22-1.53) | 1.02 (0.88-1.18) 0.788 | 0.05 (-0.13-0.22) |
| TyG tertiles 3 | 1.36 (1.28-1.44) | 1.07 (0.99-1.16) 0.075 | **0.14 (0.05-0.23)** | 1.72 (1.54-1.91) | 1.12 (0.97-1.28) 0.123 | **0.23 (0.06-0.40)** |
| **PHQ-4≥3** |  |  |  |  |  |  |
| TyG tertiles 1 | 1.00 (reference) |  |  | 1.00 (reference) |  |  |
| TyG tertiles 2 | 1.13 (1.05-1.22) | 0.99 (0.90-1.09) 0.857 | 0.04 (-0.09-0.16) | 1.15 (1.00-1.32) | 0.85 (0.71-1.00) 0.056 | -0.15 (-0.39-0.09) |
| TyG tertiles 3 | 1.38 (1.29-1.49) | 1.08 (0.99-1.18) 0.094 | **0.25 (0.13-0.37)** | 1.64 (1.44-1.86) | 1.05 (0.90-1.23) 0.540 | **0.34 (0.11-0.58)** |

TyG, triglyceride-glucose; PHQ-4, 4-item Patient Health Questionnaire; CVD, cardiovascular disease; MI, myocardial infarction.

^a^ Multivariate adjusted hazard ratios were adjusted for age, sex, race, educational level, employment status, Townsend Deprivation Index, smoking status, alcohol consumption, physical activity, diet, and self-reported medication use (including antihypertensive drugs, lipid-lowering drugs, or insulin). ^b^ Multiplicative interaction was evaluated using hazard ratios for the product term between TyG (tertiles) and PHQ-4 status (0, 1, and ≥2), and the multiplicative interaction was statistically significant when its confidence interval did not include 1. ^c^ Additive interaction was evaluated using relative excess risk due to interaction between TyG (tertiles) and PHQ-4 status (0, 1, and ≥2), and the additive interaction was statistically significant when its confidence interval did not include 0.

**Supplementary Table 12.** **Associations of PHQ-4 with incident CVD and MI and mediation proportion attributed to TyG stratified by age**

|  | **Age<65** | | **Age≥65** | |
| --- | --- | --- | --- | --- |
|  | **Multivariate adjusted hazard ratio^a^ (95% CI)** | **Mediation proportion^b^**  **(95% CI)** | **Multivariate adjusted hazard ratio^a^ (95% CI)** | **Mediation proportion^b^**  **(95% CI)** |
| **Incident CVD** |  |  |  |  |
| PHQ-4 (per1-score) | 1.04 (1.03-1.05) | 0.092 (0.075-0.117) | 1.07 (1.06-1.08) | 0.019 (0.009-0.030) |
| PHQ-4 |  |  |  |  |
| 0 | 1.00 (reference) | - | 1.00 (reference) |  |
| 1 | 1.11 (1.06-1.15) | 0.150 (-0.555-1.097) | 1.21 (1.14-1.27) | 0.008 (-0.005-0.026) |
| ≥2 | 1.21 (1.16-1.25) | **0.078 (0.059-0.105)** | 1.29 (1.22-1.36) | **0.024 (0.012-0.042)** |
| **Incident MI** |  |  |  |  |
| PHQ-4 (per1-score) | 1.04 (1.03-1.05) | 0.130 (0.095-0.187) | 1.06 (1.04-1.08) | 0.061 (0.037-0.105) |
| PHQ-4 |  |  |  |  |
| 0 | 1.00 (reference) | - | 1.00 (reference) |  |
| 1 | 1.06 (0.99-1.14) | -0.127 (-0.676-0.949) | 1.14 (1.03-1.26) | 0.036 (-0.410-0.581) |
| ≥2 | 1.17 (1.10-1.24) | **0.123 (0.086-0.232)** | 1.24 (1.12-1.37) | **0.072 (0.040-0.163)** |

TyG, triglyceride-glucose; PHQ-4, 4-item Patient Health Questionnaire; CVD, cardiovascular disease; MI, myocardial infarction

^a^ Multivariate adjusted hazard ratios were adjusted for sex, race, educational level, employment status, Townsend Deprivation Index, smoking status, alcohol consumption, physical activity, diet, and self-reported medication use (including antihypertensive drugs, lipid-lowering drugs, or insulin). ^b^ In the mediation analysis, PHQ-4 =0 was set as the reference group, and the mediation proportion was statistically significant when its confidence interval did not include 0.

**Supplementary Table 13.** **Associations of PHQ-4 with incident CVD and MI and mediation proportion attributed to TyG stratified by sex**

|  | **Female** | | **Male** | |
| --- | --- | --- | --- | --- |
|  | **Multivariate adjusted hazard ratio^a^ (95% CI)** | **Mediation proportion^b^**  **(95% CI)** | **Multivariate adjusted hazard ratio^a^ (95% CI)** | **Mediation proportion^b^**  **(95% CI)** |
| **Incident CVD** |  |  |  |  |
| PHQ-4 (per1-score) | 1.09 (1.08-1.10) | 0.055 (0.043-0.066) | 1.06 (1.06-1.07) | 0.046 (0.036-0.058) |
| PHQ-4 |  |  |  |  |
| 0 | 1.00 (reference) | - | 1.00 (reference) |  |
| 1 | 1.22 (1.15-1.28) | -0.058 (-0.529-0.692) | 1.16 (1.11-1.21) | 0.049 (-0.003-0.204) |
| ≥2 | 1.42 (1.35-1.50) | **0.056 (0.044-0.072)** | 1.30 (1.25-1.35) | **0.045 (0.034-0.058)** |
| **Incident MI** |  |  |  |  |
| PHQ-4 (per1-score) | 1.08 (1.06-1.10) | 0.093 (0.068-0.127) | 1.06 (1.05-1.08) | 0.081 (0.064-0.109) |
| PHQ-4 |  |  |  |  |
| 0 | 1.00 (reference) | - | 1.00 (reference) |  |
| 1 | 1.02 (0.91-1.13) | 0.015 (-0.029-0.065) | 1.17 (1.09-1.25) | 0.073 (-0.473-0.599) |
| ≥2 | 1.27 (1.15-1.39) | **0.099 (0.070-0.151)** | 1.30 (1.22-1.38) | **0.081 (0.060-0.110)** |

TyG, triglyceride-glucose; PHQ-4, 4-item Patient Health Questionnaire; CVD, cardiovascular disease; MI, myocardial infarction

^a^ Multivariate adjusted hazard ratios were adjusted for age, race, educational level, employment status, Townsend Deprivation Index, smoking status, alcohol consumption, physical activity, diet, and self-reported medication use (including antihypertensive drugs, lipid-lowering drugs, or insulin). ^b^ In the mediation analysis, PHQ-4 =0 was set as the reference group, and the mediation proportion was statistically significant when its confidence interval did not include 0.

**Supplementary Table 14. Associations of PHQ-4 with incident CVD and MI and mediation proportion attributed to TyG stratified by race**

|  | **Non-white** **people** | | **White people** | |
| --- | --- | --- | --- | --- |
|  | **Multivariate adjusted hazard ratio^a^ (95% CI)** | **Mediation proportion^b^**  **(95% CI)** | **Multivariate adjusted hazard ratio^a^ (95% CI)** | **Mediation proportion^b^**  **(95% CI)** |
| **Incident CVD** |  |  |  |  |
| PHQ-4 (per1-score) | 1.06 (1.04-1.09) | 0.071 (0.035-0.144) | 1.08 (1.07-1.08) | 0.054 (0.046-0.062) |
| PHQ-4 |  |  |  |  |
| 0 | 1.00 (reference) | - | 1.00 (reference) |  |
| 1 | 1.22 (0.98-1.53) | -0.054 (-0.517-0.518) | 1.18 (1.14-1.22) | 0.025 (-0.015-0.152) |
| ≥2 | 1.53 (1.29-1.81) | **0.056 (0.024-0.110)** | 1.35 (1.30-1.39) | **0.055 (0.046-0.063)** |
| **Incident MI** |  |  |  |  |
| PHQ-4 (per1-score) | 1.07 (1.02-1.11) | 0.095 (0.039-0.223) | 1.07 (1.06-1.08) | 0.094 (0.076-0.114) |
| PHQ-4 |  |  |  |  |
| 0 | 1.00 (reference) | - | 1.00 (reference) |  |
| 1 | 1.34 (0.93-1.92) | 0.139 (-0.525-0.632) | 1.12 (1.05-1.19) | 1.799 (-0.672-0.804)^c^ |
| ≥2 | 1.62 (1.22-2.14) | **0.074 (0.032-0.201)** | 1.29 (1.22-1.36) | **0.097 (0.077-0.125)** |

TyG, triglyceride-glucose; PHQ-4, 4-item Patient Health Questionnaire; CVD, cardiovascular disease; MI, myocardial infarction

^a^ Multivariate adjusted hazard ratios were adjusted for age, sex, educational level, employment status, Townsend Deprivation Index, smoking status, alcohol consumption, physical activity, diet, and self-reported medication use (including antihypertensive drugs, lipid-lowering drugs, or insulin). ^b^ In the mediation analysis, PHQ-4 =0 was set as the reference group, and the mediation proportion was statistically significant when its confidence interval did not include 0. ^c^ For the PHQ-4 = 1 vs 0 stratum, the proportion mediated exceeds 1.0 due to inconsistent mediation (the direct and indirect effects have opposite signs).

**Supplementary Table 15.** **Associations of TyG index with incident CVD by PHQ-4 status stratified by age**

|  | **Age<65** | | | **Age≥65** | | |
| --- | --- | --- | --- | --- | --- | --- |
|  | **Multivariate adjusted hazard ratio^a^ (95% CI)** | **HR for product term,**  ***P* for interaction^b^** | **Relative excess risk due to interaction^c^** | **Multivariate adjusted hazard ratio^a^ (95% CI)** | **HR for product term,**  ***P* for interaction^b^** | **Relative excess risk due to interaction^c^** |
| **PHQ-4=0** |  |  |  |  |  |  |
| TyG tertiles 1 | 1.00 (reference) |  |  | 1.00 (reference) |  |  |
| TyG tertiles 2 | 1.30 (1.21-1.40) |  |  | 1.00 (0.92-1.10) |  |  |
| TyG tertiles 3 | 1.46 (1.36-1.56) |  |  | 1.16 (1.06-1.26) |  |  |
| **PHQ-4=1** |  |  |  |  |  |  |
| TyG tertiles 1 | 1.00 (reference) |  |  | 1.00 (reference) |  |  |
| TyG tertiles 2 | 1.31 (1.20-1.43) | 1.03 (0.92-1.15) 0.608 | 0.06 (-0.08-0.19) | 0.95 (0.84-1.07) | 0.95 (0.82-1.11) 0.530 | -0.06 (-0.23-0.11) |
| TyG tertiles 3 | 1.52 (1.40-1.66) | 1.09 (0.97-1.21) 0.136 | 0.15 (0.02-0.28) | 1.11 (0.99-1.25) | 0.97 (0.84-1.11) 0.640 | -0.01 (-0.18-0.16) |
| **PHQ-4≥2** |  |  |  |  |  |  |
| TyG tertiles 1 | 1.00 (reference) |  |  | 1.00 (reference) |  |  |
| TyG tertiles 2 | 1.28 (1.19-1.38) | 0.99 (0.90-1.10) 0.891 | 0.03 (-0.10-0.15) | 1.03 (0.92-1.16) | 1.04 (0.90-1.21) 0.566 | 0.06 (-0.12-0.24) |
| TyG tertiles 3 | 1.63 (1.52-1.75) | **1.14 (1.04-1.25) 0.006** | **0.28 (0.17-0.40)** | 1.03 (0.92-1.15) | 0.91 (0.79-1.05) 0.202 | -0.09 (-0.26-0.09) |

TyG, triglyceride-glucose; PHQ-4, 4-item Patient Health Questionnaire; CVD, cardiovascular disease.

^a^ Multivariate adjusted hazard ratios were adjusted for sex, race, educational level, employment status, Townsend Deprivation Index, smoking status, alcohol consumption, physical activity, diet, and self-reported medication use (including antihypertensive drugs, lipid-lowering drugs, or insulin). ^b^ Multiplicative interaction was evaluated using hazard ratios for the product term between TyG (tertiles) and PHQ-4 status (0, 1, and ≥2), and the multiplicative interaction was statistically significant when its confidence interval did not include 1. ^c^ Additive interaction was evaluated using relative excess risk due to interaction between TyG (tertiles) and PHQ-4 status (0, 1, and ≥2), and the additive interaction was statistically significant when its confidence interval did not include 0.

**Supplementary Table 16. Associations of TyG index with incident MI by PHQ-4 status stratified by age**

|  | **Age<65** | | | **Age≥65** | | |
| --- | --- | --- | --- | --- | --- | --- |
|  | **Multivariate**  **adjusted hazard ratio^a^ (95% CI)** | **HR for product term,**  ***P* for interaction^b^** | **Relative excess risk due to interaction^c^** | **Multivariate**  **adjusted hazard ratio^a^ (95% CI)** | **HR for product term,**  ***P* for interaction^b^** | **Relative excess risk due to interaction^c^** |
| **PHQ-4=0** |  |  |  |  |  |  |
| TyG tertiles 1 | 1.00 (reference) |  |  | 1.00 (reference) |  |  |
| TyG tertiles 2 | 1.55 (1.37-1.75) |  |  | 1.20 (1.02-1.42) |  |  |
| TyG tertiles 3 | 1.78 (1.57-2.00) |  |  | 1.46 (1.24-1.71) |  |  |
| **PHQ-4=1** |  |  |  |  |  |  |
| TyG tertiles 1 | 1.00 (reference) |  |  | 1.00 (reference) |  |  |
| TyG tertiles 2 | 1.53 (1.30-1.81) | 1.03 (0.84-1.26) 0.791 | -0.01 (-0.26-0.25) | 1.13 (0.89-1.43) | 0.95 (0.72-1.27) 0.733 | -0.03 (-0.37-0.31) |
| TyG tertiles 3 | 1.92 (1.64-2.25) | 1.17 (0.96-1.42) 0.115 | 0.22 (-0.02-0.47) | 1.36 (1.09-1.70) | 0.96 (0.73-1.25) 0.739 | 0.00 (-0.34-0.34) |
| **PHQ-4≥2** |  |  |  |  |  |  |
| TyG tertiles 1 | 1.00 (reference) |  |  | 1.00 (reference) |  |  |
| TyG tertiles 2 | 1.29 (1.13-1.47) | 0.85 (0.71-1.02) 0.086 | -0.18 (-0.41-0.06) | 1.23 (0.98-1.55) | 1.04 (0.78-1.38) 0.802 | 0.10 (-0.25-0.45) |
| TyG tertiles 3 | 1.86 (1.64-2.10) | 1.10 (0.94-1.30) 0.240 | **0.31 (0.09-0.53)** | 1.34 (1.07-1.67) | 0.94 (0.72-1.23) 0.657 | 0.00 (-0.35-0.35) |

TyG, triglyceride-glucose; PHQ-4, 4-item Patient Health Questionnaire; MI, myocardial infarction.

^a^ Multivariate adjusted hazard ratios were adjusted for sex, race, educational level, employment status, Townsend Deprivation Index, smoking status, alcohol consumption, physical activity, diet, and self-reported medication use (including antihypertensive drugs, lipid-lowering drugs, or insulin). ^b^ Multiplicative interaction was evaluated using hazard ratios for the product term between TyG (tertiles) and PHQ-4 status (0, 1, and ≥2), and the multiplicative interaction was statistically significant when its confidence interval did not include 1. ^c^ Additive interaction was evaluated using relative excess risk due to interaction between TyG (tertiles) and PHQ-4 status (0, 1, and ≥2), and the additive interaction was statistically significant when its confidence interval did not include 0.

**Supplementary Table 17. Associations of TyG index with incident CVD by PHQ-4 status stratified by sex**

|  | **Female** | | | **Male** | | |
| --- | --- | --- | --- | --- | --- | --- |
|  | **Multivariate**  **adjusted hazard ratio^a^ (95% CI)** | **HR for product term,**  ***P* for interaction^b^** | **Relative excess risk due to interaction^c^** | **Multivariate**  **adjusted hazard ratio^a^ (95% CI)** | **HR for product term,**  ***P* for interaction^b^** | **Relative excess risk due to interaction^c^** |
| **PHQ-4=0** |  |  |  |  |  |  |
| TyG tertiles 1 | 1.00 (reference) |  |  | 1.00 (reference) |  |  |
| TyG tertiles 2 | 1.21 (1.10-1.33) |  |  | 1.08 (1.01-1.16) |  |  |
| TyG tertiles 3 | 1.42 (1.29-1.56) |  |  | 1.21 (1.13-1.29) |  |  |
| **PHQ-4=1** |  |  |  |  |  |  |
| TyG tertiles 1 | 1.00 (reference) |  |  | 1.00 (reference) |  |  |
| TyG tertiles 2 | 1.10 (0.98-1.22) | 0.93 (0.81-1.07) 0.316 | -0.05 (-0.22-0.13) | 1.10 (1.00-1.22) | 1.03 (0.92-1.16) 0.608 | 0.04 (-0.09-0.18) |
| TyG tertiles 3 | 1.22 (1.09-1.36) | 0.91(0.79-1.04) 0.164 | -0.06 (-0.24-0.13) | 1.32 (1.20-1.45) | 1.11 (0.99-1.24) 0.071 | 0.16 (0.03-0.28) |
| **PHQ-4≥2** |  |  |  |  |  |  |
| TyG tertiles 1 | 1.00 (reference) |  |  | 1.00 (reference) |  |  |
| TyG tertiles 2 | 1.19 (1.08-1.30) | 0.98 (0.86-1.12) 0.777 | 0.05 (-0.12-0.21) | 1.10 (1.01-1.20) | 1.03 (0.92-1.15) 0.637 | 0.05 (-0.08-0.18) |
| TyG tertiles 3 | 1.47 (1.34-1.60) | 1.05 (0.93-1.19) 0.409 | **0.26 (0.08-0.43)** | 1.30 (1.20-1.41) | 1.11 (1.00-1.22) 0.053 | **0.20 (0.08-0.32)** |

TyG, triglyceride-glucose; PHQ-4, 4-item Patient Health Questionnaire; CVD, cardiovascular disease.

^a^ Multivariate adjusted hazard ratios were adjusted for age, race, educational level, employment status, Townsend Deprivation Index, smoking status, alcohol consumption, physical activity, diet, and self-reported medication use (including antihypertensive drugs, lipid-lowering drugs, or insulin). ^b^ Multiplicative interaction was evaluated using hazard ratios for the product term between TyG (tertiles) and PHQ-4 status (0, 1, and ≥2), and the multiplicative interaction was statistically significant when its confidence interval did not include 1. ^c^ Additive interaction was evaluated using relative excess risk due to interaction between TyG (tertiles) and PHQ-4 status (0, 1, and ≥2), and the additive interaction was statistically significant when its confidence interval did not include 0.

**Supplementary Table 18. Associations of TyG index with incident MI by PHQ-4 status stratified by sex**

|  | **Female** | | | **Male** | | |
| --- | --- | --- | --- | --- | --- | --- |
|  | **Multivariate**  **adjusted hazard ratio^a^ (95% CI)** | **HR for product term,**  ***P* for interaction^b^** | **Relative excess risk due to interaction^c^** | **Multivariate**  **adjusted hazard ratio^a^ (95% CI)** | **HR for product term,**  ***P* for interaction^b^** | **Relative excess risk due to interaction^c^** |
| **PHQ-4=0** |  |  |  |  |  |  |
| TyG tertiles 1 | 1.00 (reference) |  |  | 1.00 (reference) |  |  |
| TyG tertiles 2 | 1.38 (1.15-1.65) |  |  | 1.34 (1.19-1.51) |  |  |
| TyG tertiles 3 | 1.71 (1.43-2.05) |  |  | 1.54 (1.37-1.72) |  |  |
| **PHQ-4=1** |  |  |  |  |  |  |
| TyG tertiles 1 | 1.00 (reference) |  |  | 1.00 (reference) |  |  |
| TyG tertiles 2 | 1.36 (1.09-1.70) | 1.01 (0.76-1.34) 0.952 | 0.03 (-0.30-0.37) | 1.29 (1.08-1.53) | 0.97 (0.79-1.19) 0.765 | -0.01 (-0.26-0.24) |
| TyG tertiles 3 | 1.47 (1.17-1.84) | 0.89 (0.67-1.18) 0.418 | -0.16 (-0.53-0.21) | 1.70 (1.45-2.00) | 1.13 (0.93-1.37) 0.226 | 0.26 (0.03-0.50) |
| **PHQ-4≥2** |  |  |  |  |  |  |
| TyG tertiles 1 | 1.00 (reference) |  |  | 1.00 (reference) |  |  |
| TyG tertiles 2 | 1.17 (0.98-1.41) | 0.86 (0.66-1.10) 0.226 | -0.15 (-0.48-0.18) | 1.22 (1.06-1.42) | 0.93 (0.77-1.12) 0.419 | -0.04 (-0.28-0.21) |
| TyG tertiles 3 | 1.74 (1.47-2.07) | 1.06 (0.83-1.34) 0.663 | 0.30 (-0.04-0.65) | 1.58 (1.38-1.80) | 1.06 (0.89-1.27) 0.491 | **0.26 (0.03-0.49)** |

TyG, triglyceride-glucose; PHQ-4, 4-item Patient Health Questionnaire; MI, myocardial infarction.

^a^ Multivariate adjusted hazard ratios were adjusted for age, race, educational level, employment status, Townsend Deprivation Index, smoking status, alcohol consumption, physical activity, diet, and self-reported medication use (including antihypertensive drugs, lipid-lowering drugs, or insulin). ^b^ Multiplicative interaction was evaluated using hazard ratios for the product term between TyG (tertiles) and PHQ-4 status (0, 1, and ≥2), and the multiplicative interaction was statistically significant when its confidence interval did not include 1. ^c^ Additive interaction was evaluated using relative excess risk due to interaction between TyG (tertiles) and PHQ-4 status (0, 1, and ≥2), and the additive interaction was statistically significant when its confidence interval did not include 0.

**Supplementary Table 19.** **Associations of TyG index with incident CVD by PHQ-4 status stratified by race**

|  | **Non-white people** | | | **White people** | | |
| --- | --- | --- | --- | --- | --- | --- |
|  | **Multivariate**  **adjusted hazard ratio^a^ (95% CI)** | **HR for product term,**  ***P* for interaction^b^** | **Relative excess risk due to interaction^c^** | **Multivariate**  **adjusted hazard ratio^a^ (95% CI)** | **HR for product term,**  ***P* for interaction^b^** | **Relative excess risk due to interaction^c^** |
| **PHQ-4=0** |  |  |  |  |  |  |
| TyG tertiles 1 | 1.00 (reference) |  |  | 1.00 (reference) |  |  |
| TyG tertiles 2 | 1.36 (0.98-1.89) |  |  | 1.14 (1.07-1.20) |  |  |
| TyG tertiles 3 | 1.38 (1.00-1.91) |  |  | 1.30 (1.23-1.37) |  |  |
| **PHQ-4=1** |  |  |  |  |  |  |
| TyG tertiles 1 | 1.00 (reference) |  |  | 1.00 (reference) |  |  |
| TyG tertiles 2 | 0.88 (0.53-1.48) | 0.64 (0.35-1.17) 0.146 | -0.53 (-1.29-0.24) | 1.13 (1.05-1.21) | 1.01 (0.92-1.10) 0.886 | 0.03 (-0.08-0.13) |
| TyG tertiles 3 | 1.82 (1.17-2.81) | 1.20(0.71-2.04) 0.488 | 0.46 (-0.30-1.22) | 1.31 (1.22-1.41) | 1.04 (0.95-1.13) 0.420 | 0.10 (-0.01-0.20) |
| **PHQ-4≥2** |  |  |  |  |  |  |
| TyG tertiles 1 | 1.00 (reference) |  |  | 1.00 (reference) |  |  |
| TyG tertiles 2 | 1.21 (0.93-1.58) | 0.90 (0.59-1.36) 0.609 | -0.01 (-0.61-0.59) | 1.17 (1.09-1.24) | 1.03 (0.95-1.12) 0.466 | 0.08 (-0.02-0.18) |
| TyG tertiles 3 | 1.30 (1.01-1.67) | 0.96 (0.64-1.44) 0.858 | 0.14 (-0.43-0.72) | 1.42 (1.34-1.51) | **1.12 (1.03-1.21) 0.007** | **0.26 (0.16-0.36)** |

TyG, triglyceride-glucose; PHQ-4, 4-item Patient Health Questionnaire; CVD, cardiovascular disease.

^a^ Multivariate adjusted hazard ratios were adjusted for age, sex, educational level, employment status, Townsend Deprivation Index, smoking status, alcohol consumption, physical activity, diet, and self-reported medication use (including antihypertensive drugs, lipid-lowering drugs, or insulin). ^b^ Multiplicative interaction was evaluated using hazard ratios for the product term between TyG (tertiles) and PHQ-4 status (0, 1, and ≥2), and the multiplicative interaction was statistically significant when its confidence interval did not include 1. ^c^ Additive interaction was evaluated using relative excess risk due to interaction between TyG (tertiles) and PHQ-4 status (0, 1, and ≥2), and the additive interaction was statistically significant when its confidence interval did not include 0.

**Supplementary Table 20.** **Associations of TyG index with incident MI by PHQ-4 status stratified by race**

|  | **Non-white people** | | | **White people** | | |
| --- | --- | --- | --- | --- | --- | --- |
|  | **Multivariate**  **adjusted hazard ratio^a^ (95% CI)** | **HR for product term,**  ***P* for interaction^b^** | **Relative excess risk due to interaction^c^** | **Multivariate**  **adjusted hazard ratio^a^ (95% CI)** | **HR for product term,**  ***P* for interaction^b^** | **Relative excess risk due to interaction^c^** |
| **PHQ-4=0** |  |  |  |  |  |  |
| TyG tertiles 1 | 1.00 (reference) |  |  | 1.00 (reference) |  |  |
| TyG tertiles 2 | 1.92 (1.07-3.45) |  |  | 1.37 (1.24-1.51) |  |  |
| TyG tertiles 3 | 1.82 (1.01-3.25) |  |  | 1.62 (1.47-1.79) |  |  |
| **PHQ-4=1** |  |  |  |  |  |  |
| TyG tertiles 1 | 1.00 (reference) |  |  | 1.00 (reference) |  |  |
| TyG tertiles 2 | 0.99 (0.44-2.21) | 0.52 (0.19-1.38) 0.186 | -0.93 (-2.74-0.87) | 1.36 (1.19-1.56) | 1.02 (0.86-1.21) 0.800 | 0.05 (-0.15-0.25) |
| TyG tertiles 3 | 1.36 (0.68-2.74) | 0.75 (0.31-1.82) 0.522 | -0.17 (-1.81-1.48) | 1.71 (1.50-1.95) | 1.11 (0.95-1.30) 0.203 | 0.21 (0.01-0.41) |
| **PHQ-4≥2** |  |  |  |  |  |  |
| TyG tertiles 1 | 1.00 (reference) |  |  | 1.00 (reference) |  |  |
| TyG tertiles 2 | 1.69 (1.06-2.71) | 0.91 (0.43-1.91) 0.800 | 0.26 (-0.98-1.49) | 1.23 (1.09-1.38) | 0.91 (0.78-1.07) 0.248 | -0.06 (-0.26-0.14) |
| TyG tertiles 3 | 1.80 (1.14-2.84) | 1.02 (0.50-2.11) 0.949 | 0.55 (-0.58-1.69) | 1.70 (1.53-1.90) | 1.10 (0.95-1.27) 0.198 | **0.33 (0.14-0.52)** |

TyG, triglyceride-glucose; PHQ-4, 4-item Patient Health Questionnaire; MI, myocardial infarction.

^a^ Multivariate adjusted hazard ratios were adjusted for age, sex, educational level, employment status, Townsend Deprivation Index, smoking status, alcohol consumption, physical activity, diet, and self-reported medication use (including antihypertensive drugs, lipid-lowering drugs, or insulin). ^b^ Multiplicative interaction was evaluated using hazard ratios for the product term between TyG (tertiles) and PHQ-4 status (0, 1, and ≥2), and the multiplicative interaction was statistically significant when its confidence interval did not include 1. ^c^ Additive interaction was evaluated using relative excess risk due to interaction between TyG (tertiles) and PHQ-4 status (0, 1, and ≥2), and the additive interaction was statistically significant when its confidence interval did not include 0.

**Supplementary Table 21.** **Joint associations of TyG index and PHQ-4 status with incident CVD and MI stratified by age**

|  | **Incident CVD** | | | **Incident MI** | | |
| --- | --- | --- | --- | --- | --- | --- |
|  | **Age<65** | **Age≥65** | ***P* for interaction** | **Age<65** | **Age≥65** | ***P* for interaction** |
| PHQ-4=0 and TyG tertile 1 | 1.00 (reference) | 1.00 (reference) |  | 1.00 (reference) | 1.00 (reference) |  |
| PHQ-4=0 and TyG tertile 2 | 1.29 (1.20-1.38) | 1.00 (0.92-1.09) | **<0.001** | 1.52 (1.34-1.72) | 1.19 (1.01-1.41) | 0.010 |
| PHQ-4=0 and TyG tertile 3 | 1.44 (1.34-1.54) | 1.15 (1.05-1.25) | **<0.001** | 1.71 (1.52-1.93) | 1.44 (1.23-1.69) | **0.013** |
| PHQ-4=1 and TyG tertile 1 | 1.05 (0.96-1.15) | 1.24 (1.11-1.39) | **0.008** | 0.97 (0.82-1.14) | 1.18 (0.94-1.48) | 0.116 |
| PHQ-4=1 and TyG tertile 2 | 1.39 (1.29-1.51) | 1.18 (1.07-1.31) | **0.007** | 1.52 (1.32-1.74) | 1.34 (1.10-1.63) | 0.262 |
| PHQ-4=1 and TyG tertile 3 | 1.64 (1.52-1.76) | 1.37 (1.25-1.51) | **<0.001** | 1.94 (1.71-2.20) | 1.62 (1.36-1.94) | **0.027** |
| PHQ-4≥2 and TyG tertile 1 | 1.12 (1.04-1.21) | 1.32 (1.17-1.48) | **0.008** | 1.15 (1.00-1.32) | 1.25 (0.99-1.57) | 0.450 |
| PHQ-4≥2 and TyG tertile 2 | 1.44 (1.34-1.54) | 1.38 (1.25-1.52) | 0.311 | 1.49 (1.31-1.70) | 1.54 (1.28-1.86) | 0.887 |
| PHQ-4≥2 and TyG tertile 3 | 1.84 (1.72-1.97) | 1.38 (1.25-1.51) | **<0.001** | 2.17 (1.93-2.43) | 1.69 (1.42-2.01) | **0.002** |

TyG, triglyceride-glucose; PHQ-4, 4-item Patient Health Questionnaire; CVD, cardiovascular disease; MI, myocardial infarction.

Hazard ratio and 95% confidence interval of outcomes were present and adjusted for sex, race, educational level, employment status, Townsend Deprivation Index, smoking status, alcohol consumption, physical activity, diet, and self-reported medication use (including antihypertensive drugs, lipid-lowering drugs, or insulin).

**Supplementary Table 22.** **Joint associations of TyG index and PHQ-4 status with incident CVD and MI stratified by sex**

|  | **Incident CVD** | | | **Incident MI** | | |
| --- | --- | --- | --- | --- | --- | --- |
|  | **Female** | **Male** | ***P* for interaction** | **Female** | **Male** | ***P* for interaction** |
| PHQ-4=0 and TyG tertile 1 | 1.00 (reference) | 1.00 (reference) |  | 1.00 (reference) | 1.00 (reference) |  |
| PHQ-4=0 and TyG tertile 2 | 1.20 (1.10-1.32) | 1.07 (1.00-1.15) | **0.010** | 1.37 (1.14-1.64) | 1.33 (1.18-1.50) | 0.398 |
| PHQ-4=0 and TyG tertile 3 | 1.39 (1.27-1.53) | 1.20 (1.12-1.28) | **<0.001** | 1.67 (1.40-2.00) | 1.51 (1.35-1.70) | **0.043** |
| PHQ-4=1 and TyG tertile 1 | 1.29 (1.16-1.43) | 1.09 (0.99-1.19) | **0.042** | 1.05 (0.84-1.31) | 1.10 (0.93-1.30) | 0.608 |
| PHQ-4=1 and TyG tertile 2 | 1.44 (1.30-1.59) | 1.20 (1.11-1.31) | **0.002** | 1.45 (1.19-1.76) | 1.42 (1.24-1.64) | 0.535 |
| PHQ-4=1 and TyG tertile 3 | 1.62 (1.47-1.79) | 1.44 (1.34-1.55) | **0.002** | 1.56 (1.29-1.90) | 1.88 (1.66-2.13) | 0.616 |
| PHQ-4≥2 and TyG tertile 1 | 1.38 (1.26-1.52) | 1.22 (1.12-1.33) | 0.162 | 1.28 (1.05-1.55) | 1.27 (1.09-1.48) | 0.804 |
| PHQ-4≥2 and TyG tertile 2 | 1.63 (1.49-1.79) | 1.35 (1.25-1.45) | **<0.001** | 1.49 (1.25-1.79) | 1.57 (1.38-1.79) | 0.952 |
| PHQ-4≥2 and TyG tertile 3 | 2.03 (1.86-2.22) | 1.61 (1.51-1.73) | **<0.001** | 2.25 (1.90-2.67) | 2.05 (1.82-2.30) | **0.032** |

TyG, triglyceride-glucose; PHQ-4, 4-item Patient Health Questionnaire; CVD, cardiovascular disease; MI, myocardial infarction.

Hazard ratio and 95% confidence interval of outcomes were present and adjusted for age, race, educational level, employment status, Townsend Deprivation Index, smoking status, alcohol consumption, physical activity, diet, and self-reported medication use (including antihypertensive drugs, lipid-lowering drugs, or insulin).

**Supplementary Table 23.** **Joint associations of TyG index and PHQ-4 status with incident CVD and MI stratified by race**

|  | **Incident CVD** | | | **Incident MI** | | |
| --- | --- | --- | --- | --- | --- | --- |
|  | **Non-white people** | **White people** | ***P* for interaction** | **Non-white people** | **White people** | ***P* for interaction** |
| PHQ-4=0 and TyG tertile 1 | 1.00 (reference) | 1.00 (reference) |  | 1.00 (reference) | 1.00 (reference) |  |
| PHQ-4=0 and TyG tertile 2 | 1.36 (0.98-1.89) | 1.13 (1.07-1.19) | 0.247 | 1.87 (1.05-3.36) | 1.35 (1.22-1.50) | 0.192 |
| PHQ-4=0 and TyG tertile 3 | 1.38 (1.00-1.91) | 1.29 (1.22-1.36) | 0.611 | 1.79 (1.01-3.18) | 1.58 (1.44-1.74) | 0.423 |
| PHQ-4=1 and TyG tertile 1 | 1.27 (0.84-1.90) | 1.15 (1.07-1.24) | 0.527 | 1.83 (0.91-3.69) | 1.05 (0.92-1.20) | 0.100 |
| PHQ-4=1 and TyG tertile 2 | 1.10 (0.69-1.74) | 1.31 (1.23-1.39) | 0.491 | 1.77 (0.84-3.76) | 1.45 (1.29-1.62) | 0.563 |
| PHQ-4=1 and TyG tertile 3 | 2.11 (1.48-3.01) | 1.53 (1.45-1.63) | 0.063 | 2.46 (1.31-4.63) | 1.84 (1.66-2.04) | 0.224 |
| PHQ-4≥2 and TyG tertile 1 | 1.58 (1.16-2.15) | 1.25 (1.17-1.34) | 0.095 | 1.61 (0.89-2.92) | 1.24 (1.10-1.41) | 0.410 |
| PHQ-4≥2 and TyG tertile 2 | 1.93 (1.43-2.61) | 1.46 (1.38-1.55) | 0.051 | 2.74 (1.59-4.73) | 1.54 (1.38-1.71) | 0.036 |
| PHQ-4≥2 and TyG tertile 3 | 2.11 (1.58-2.81) | 1.80 (1.70-1.90) | 0.175 | 2.96 (1.75-5.01) | 2.16 (1.96-2.38) | 0.148 |

TyG, triglyceride-glucose; PHQ-4, 4-item Patient Health Questionnaire; CVD, cardiovascular disease; MI, myocardial infarction.

Hazard ratio and 95% confidence interval of outcomes were present and adjusted for age, sex, educational level, employment status, Townsend Deprivation Index, smoking status, alcohol consumption, physical activity, diet, and self-reported medication use (including antihypertensive drugs, lipid-lowering drugs, or insulin).


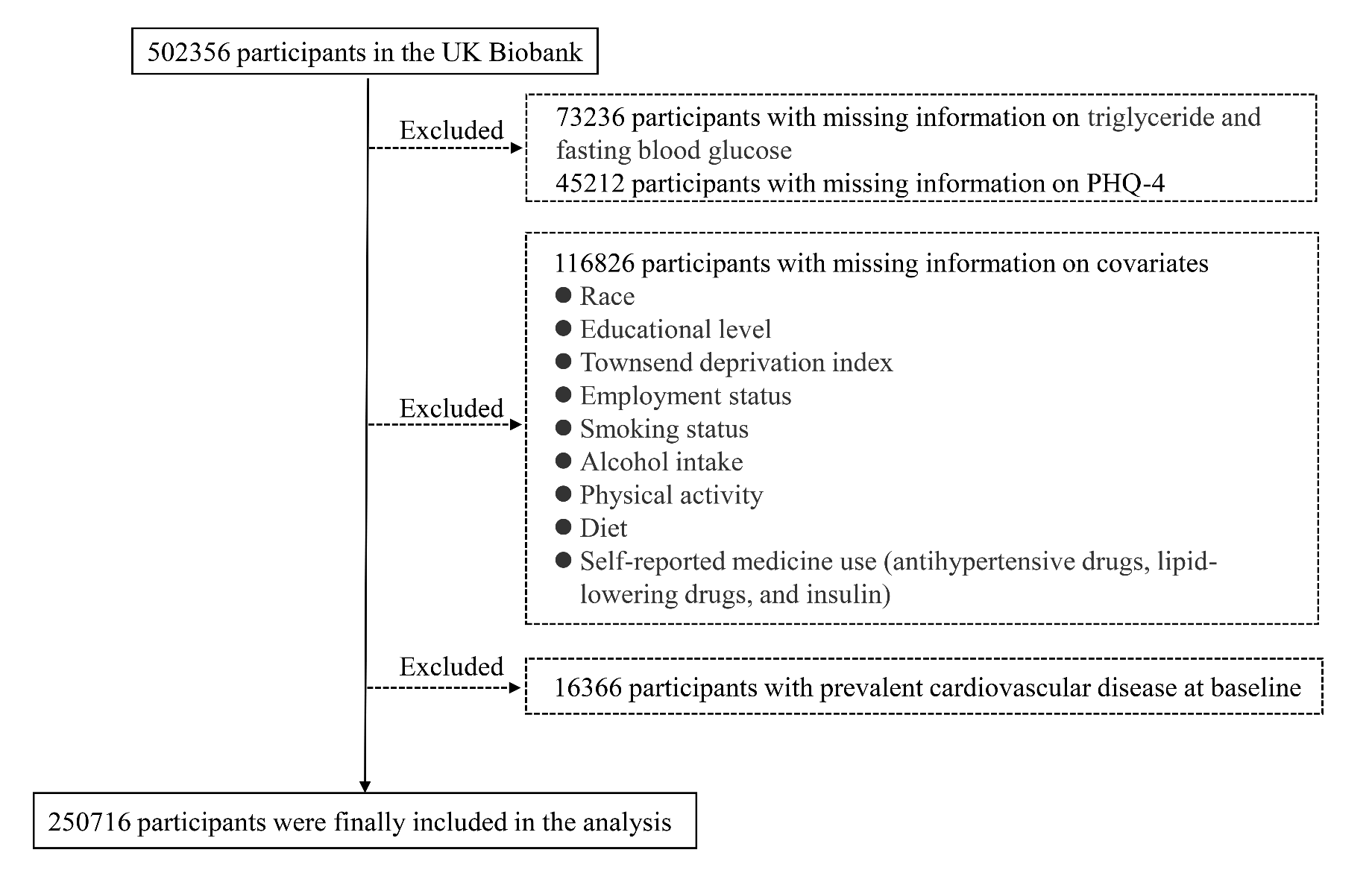


**Supplementary Figure 1.** **Flowchart of participants included in the analysis**


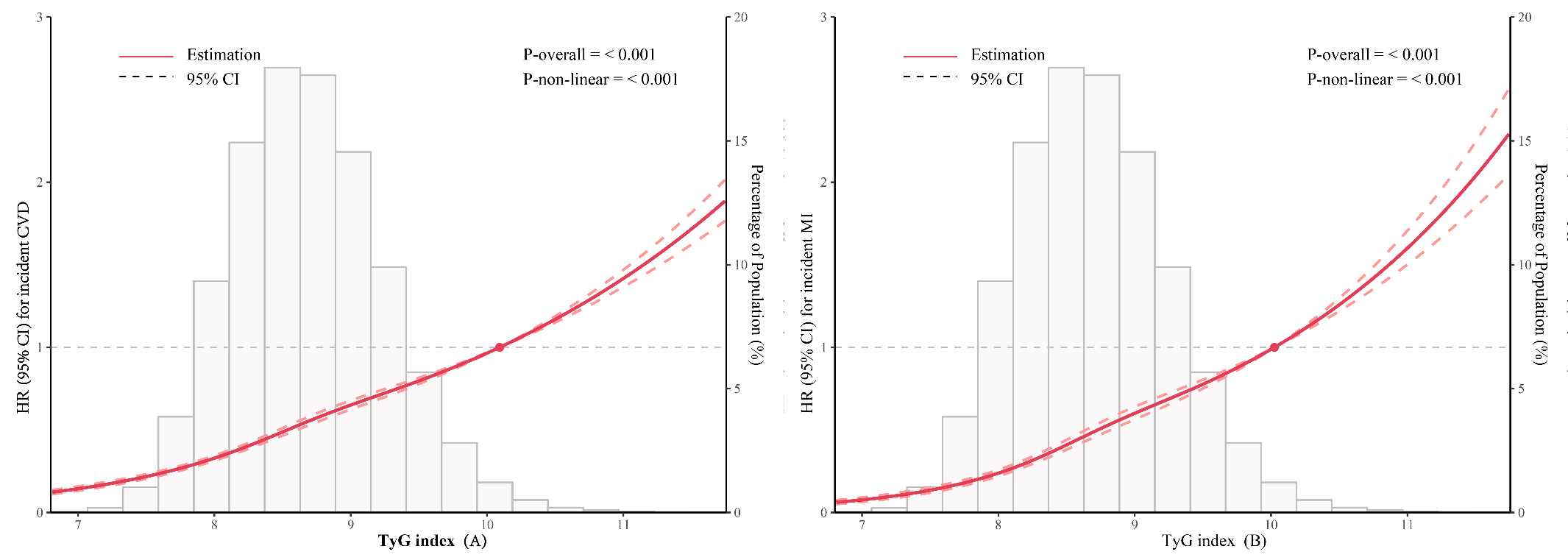


**Supplementary Figure 2.** **Dose-response associations of TyG index with incident CVD (A) and MI (B)**

TyG, triglyceride-glucose; CVD, cardiovascular disease.

Hazard ratios were adjusted for age, sex, race, educational level, employment status, Townsend Deprivation Index, smoking status, alcohol consumption, physical activity, diet, and self-reported medication use (including antihypertensive drugs, lipid-lowering drugs, or insulin).


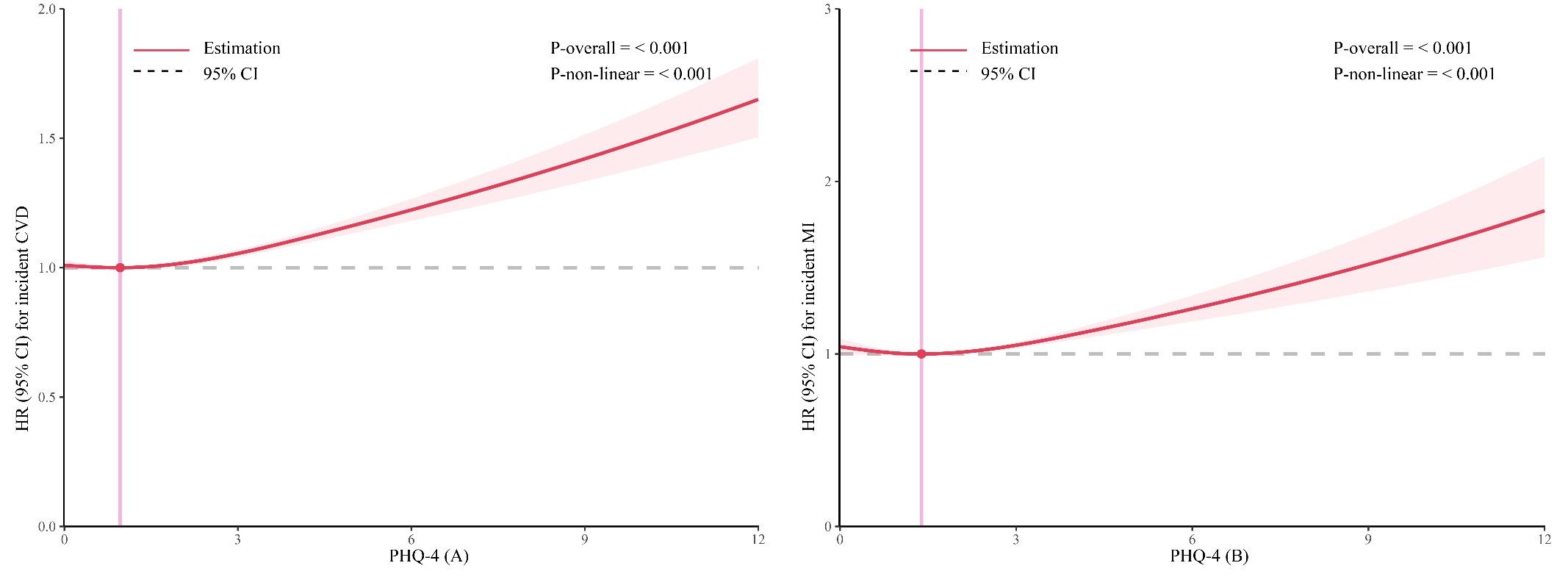


**Supplementary Figure 3.** **Dose-response associations of PHQ-4 scores with incident CVD (A) and MI (B)**

PHQ-4, 4-item Patient Health Questionnaire; MI, myocardial infarction.

Hazard ratios were adjusted for age, sex, race, educational level, employment status, Townsend Deprivation Index, smoking status, alcohol consumption, physical activity, diet, and self-reported medication use (including antihypertensive drugs, lipid-lowering drugs, or insulin).


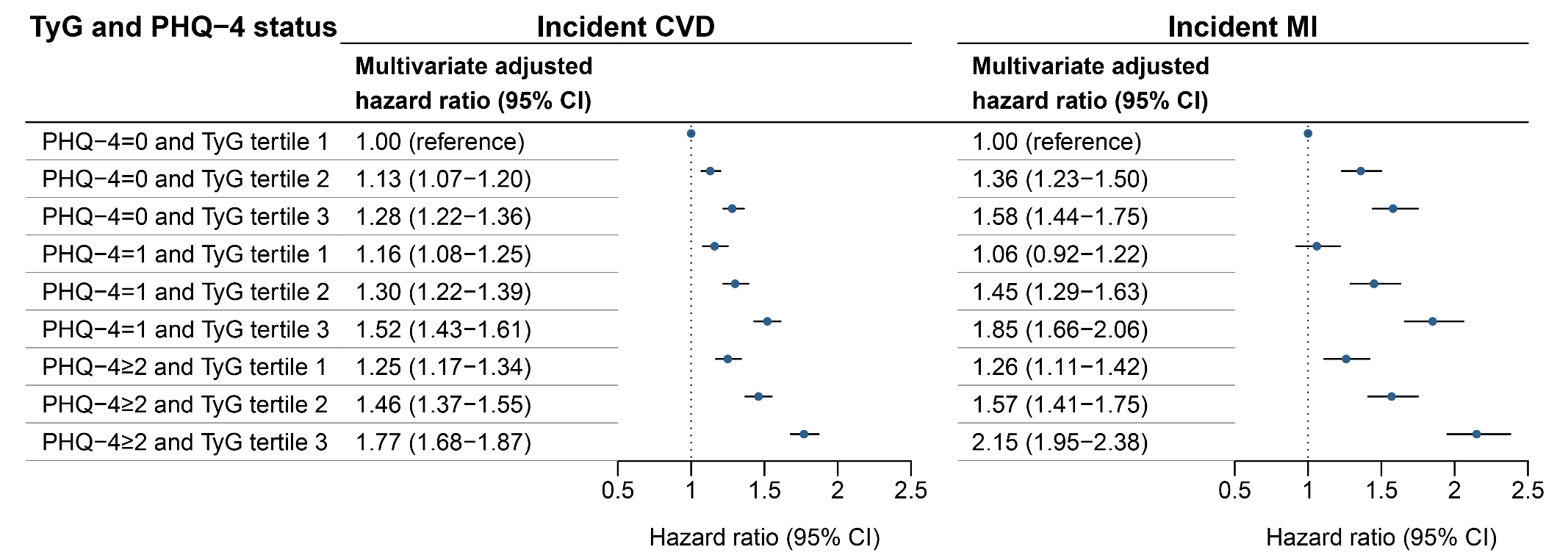


**Supplementary Figure 4.** **Joint associations of TyG index and PHQ-4 status with incident CVD and MI after excluding the cases that occurred within first 2 years of follow-up**

TyG, triglyceride-glucose; PHQ-4, 4-item Patient Health Questionnaire; CVD, cardiovascular disease; MI, myocardial infarction.

Multivariate adjusted hazard ratios were adjusted for age, sex, race, educational level, employment status, Townsend Deprivation Index, smoking status, alcohol consumption, physical activity, diet, and self-reported medication use (including antihypertensive drugs, lipid-lowering drugs, or insulin).


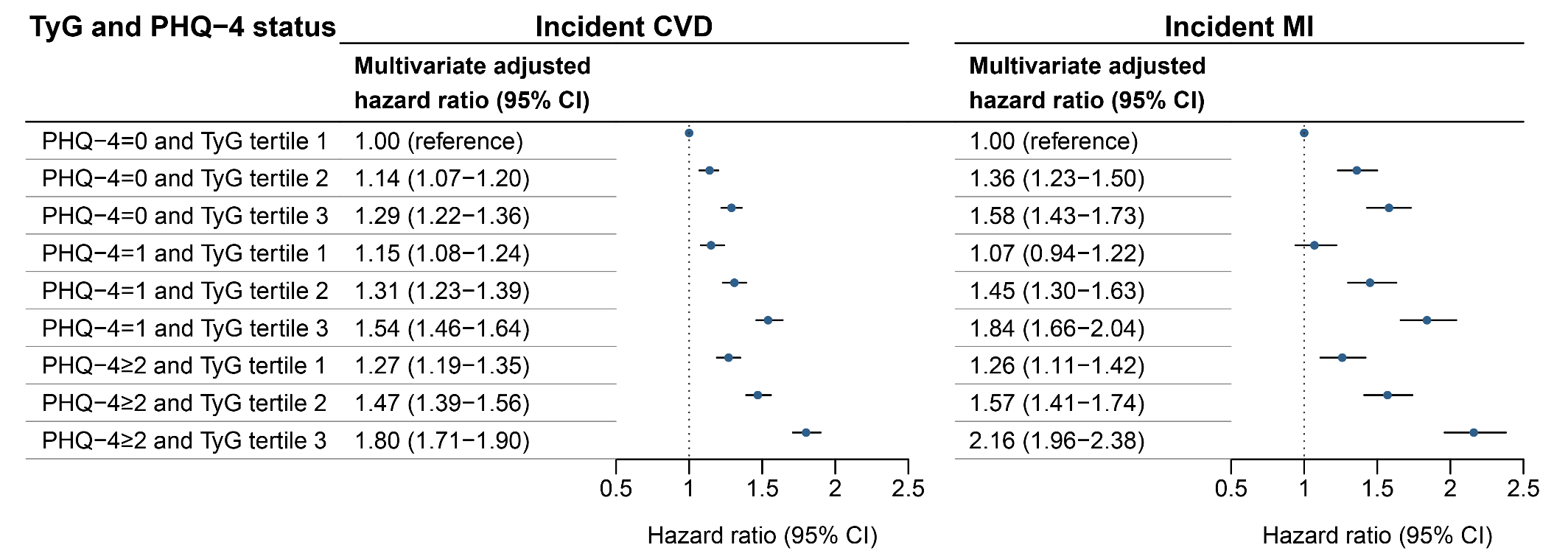


**Supplementary Figure 5.** **Joint associations of TyG index and PHQ-4 status with incident CVD and MI after further inclusion of quadratic terms of age in the model**

TyG, triglyceride-glucose; PHQ-4, 4-item Patient Health Questionnaire; CVD, cardiovascular disease; MI, myocardial infarction.

Multivariate adjusted hazard ratios were adjusted for age, age^2^, sex, race, educational level, employment status, Townsend Deprivation Index, smoking status, alcohol consumption, physical activity, diet, and self-reported medication use (including antihypertensive drugs, lipid-lowering drugs, or insulin).


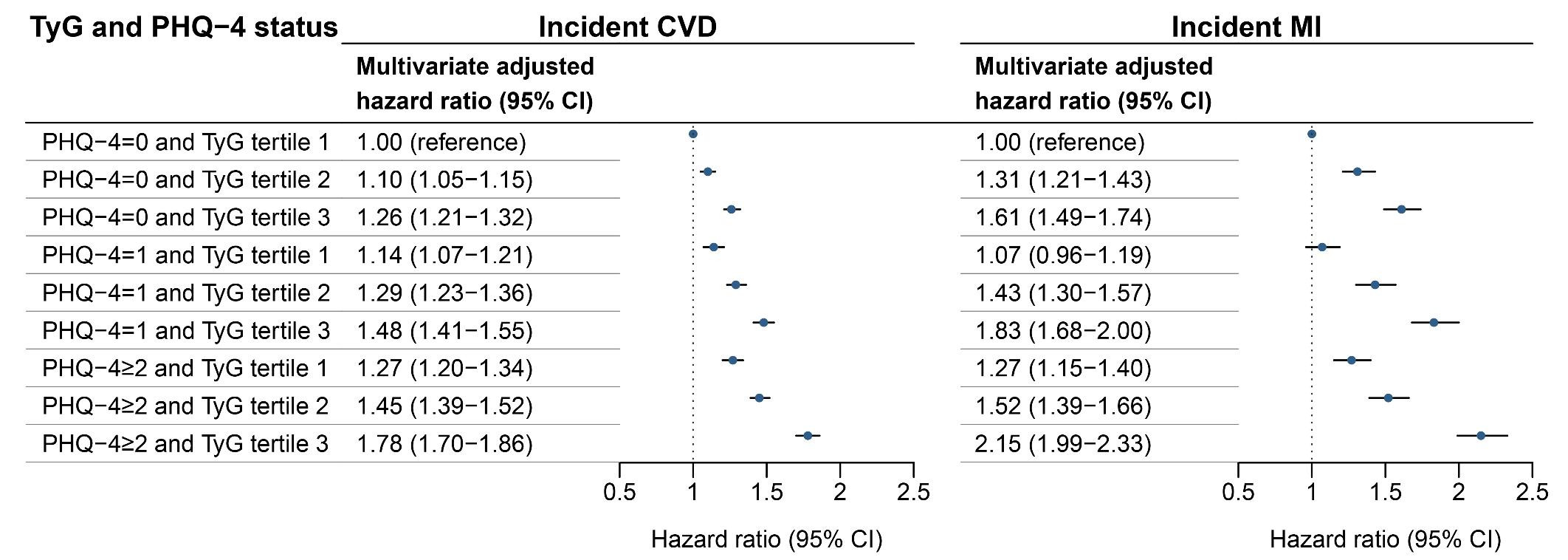


**Supplementary Figure 6.** **Joint associations of TyG index and PHQ-4 status with incident CVD and MI after using multiple imputations with chained equations**

TyG, triglyceride-glucose; PHQ-4, 4-item Patient Health Questionnaire; CVD, cardiovascular disease; MI, myocardial infarction.

Multivariate adjusted hazard ratios were adjusted for age, sex, race, educational level, employment status, Townsend Deprivation Index, smoking status, alcohol consumption, physical activity, diet, and self-reported medication use (including antihypertensive drugs, lipid-lowering drugs, or insulin).


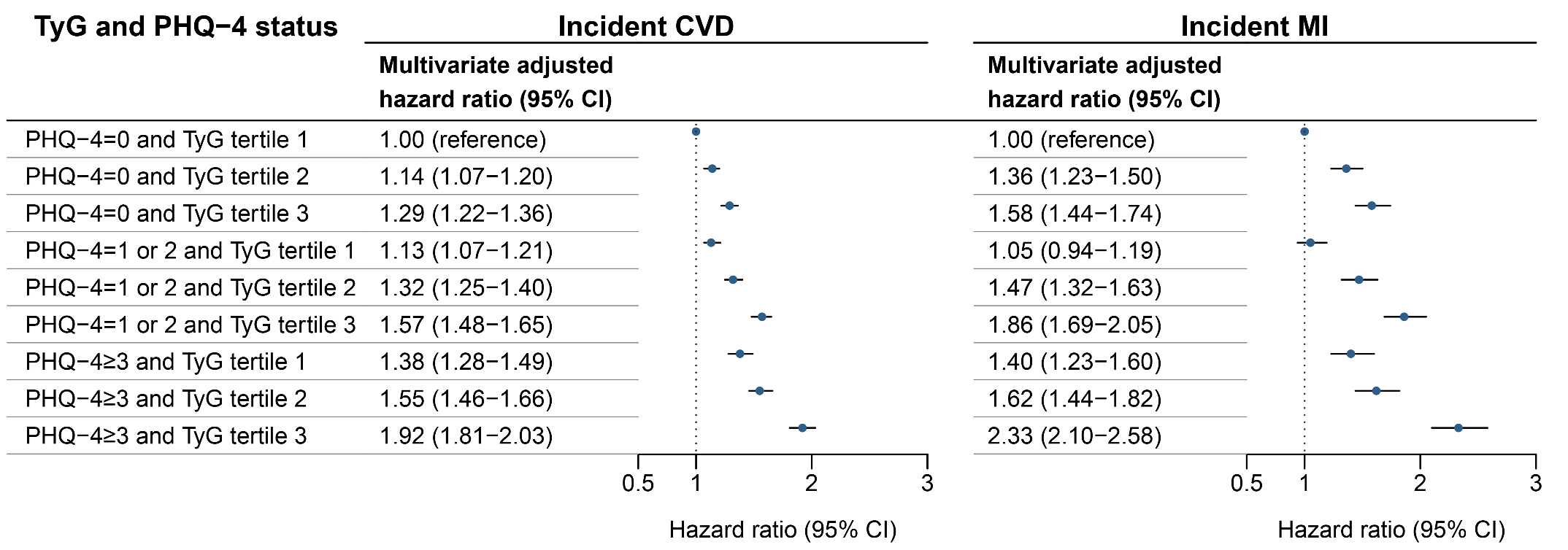


**Supplementary Figure 7.** **Joint associations of TyG index and PHQ-4 status with incident CVD and MI when using additional threshold for defining PHQ-4 status**

TyG, triglyceride-glucose; PHQ-4, 4-item Patient Health Questionnaire; CVD, cardiovascular disease; MI, myocardial infarction.

Multivariate adjusted hazard ratios were adjusted for age, sex, race, educational level, employment status, Townsend Deprivation Index, smoking status, alcohol consumption, physical activity, diet, and self-reported medication use (including antihypertensive drugs, lipid-lowering drugs, or insulin).
